# A novel RNA binding protein affects *rbc*L gene expression and is specific to bundle sheath chloroplasts in C_4_ plants

**DOI:** 10.1186/1471-2229-13-138

**Published:** 2013-09-22

**Authors:** Shaun M Bowman, Minesh Patel, Pradeep Yerramsetty, Christopher M Mure, Amy M Zielinski, Jeremy A Bruenn, James O Berry

**Affiliations:** 1 Department of Biological Sciences, University at Buffalo, Buffalo, NY 14260, USA; 2 Current Address: Biology Department, Clarke University, Dubuque, IA 52001, USA; 3 Current Address: Department of Crop Science, North Carolina State University, Raleigh, NC 27695, USA

**Keywords:** C_4_ photosynthesis, S1-domain RNA binding protein, *rbc*L gene expression, Cell-type specificity, Post transcriptional control, C_4_ maize, C_3_*Arabidopsis*

## Abstract

**Background:**

Plants that utilize the highly efficient C_4_ pathway of photosynthesis typically possess kranz-type leaf anatomy that consists of two morphologically and functionally distinct photosynthetic cell types, the bundle sheath (BS) and mesophyll (M) cells. These two cell types differentially express many genes that are required for C_4_ capability and function. In mature C_4_ leaves, the plastidic *rbc*L gene, encoding the large subunit of the primary CO_2_ fixation enzyme Rubisco, is expressed specifically within BS cells. Numerous studies have demonstrated that BS-specific *rbc*L gene expression is regulated predominantly at post-transcriptional levels, through the control of translation and mRNA stability. The identification of regulatory factors associated with C_4_ patterns of *rbc*L gene expression has been an elusive goal for many years.

**Results:**

RLSB, encoded by the nuclear *RLSB* gene, is an S1-domain RNA binding protein purified from C_4_ chloroplasts based on its specific binding to plastid-encoded *rbc*L mRNA *in vitro*. Co-localized with LSU to chloroplasts, RLSB is highly conserved across many plant species. Most significantly, RLSB localizes specifically to leaf bundle sheath (BS) cells in C_4_ plants. Comparative analysis using maize (C_4_) and *Arabidopsis* (C_3_) reveals its tight association with *rbc*L gene expression in both plants. Reduced *RLSB* expression (through insertion mutation or RNA silencing, respectively) led to reductions in *rbc*L mRNA accumulation and LSU production. Additional developmental effects, such as virescent/yellow leaves, were likely associated with decreased photosynthetic function and disruption of associated signaling networks.

**Conclusions:**

Reductions in *RLSB* expression, due to insertion mutation or gene silencing, are strictly correlated with reductions in *rbc*L gene expression in both maize and *Arabidopsis*. In both plants, accumulation of *rbc*L mRNA as well as synthesis of LSU protein were affected. These findings suggest that specific accumulation and binding of the RLSB binding protein to *rbc*L mRNA within BS chloroplasts may be one determinant leading to the characteristic cell type-specific localization of Rubisco in C_4_ plants. Evolutionary modification of *RLSB* expression, from a C_3_ “default” state to BS cell-specificity, could represent one mechanism by which *rbc*L expression has become restricted to only one cell type in C_4_ plants.

## Background

The highly efficient C_4_ pathway of photosynthetic carbon assimilation is utilized by less than 5% of terrestrial plants, and yet C_4_ plants account for about a fourth of the earth’s primary productivity [1-4]. The enhanced photosynthetic capabilities of C_4_ plant species allow them to out-compete more common and less efficient C_3_ species. This is most evident in areas of high temperature and/or low water availability, conditions under which C_4_ plants typically thrive. In spite of their much higher productivity, there are only a few C_4_ plant species utilized as crops for food and biofuel production, the most notable being maize and sugarcane [1-4]. Understanding the specialized developmental, molecular, and biochemical processes responsible for C_4_ function is a significant focus of photosynthesis and agricultural research. Agricultural benefits include contributing to the development of non-agricultural C_4_ plants that are more amenable to agricultural usage, understanding mechanisms of plant adaption to extreme arid conditions, and possibly enabling the engineering of C_4_ characteristics into C_3_ crop species [1-5]. As a unique developmental system, the specific localization of key photosynthetic enzymes to one cell type, but not in another adjacent cell type within a small localized leaf region, provides a unique opportunity to address molecular mechanisms underlying the selective compartmentalization of gene expression in plants [5,6].

Characteristics common to all C_4_ species include utilization of phosphoenolpyruvate carboxylase (PEPCase) as the initial primary CO_2_ fixation enzyme and production of C_4_ acids by a first stage of reactions, followed by decarboxylation of C_4_ acids, and subsequent re-fixation of released CO_2_ by Rubisco (Calvin cycle) in a second stage. Through the partitioning of Rubisco, C_4_ plants reduce or eliminate the photosynthetically wasteful reactions of photorespiration, thereby enhancing their CO_2_ fixation ability [3,5-9]. C_4_ plants typically possess kranz-type leaf anatomy consisting of two distinct photosynthetic cell types, bundle sheath (BS) cells and mesophyll (M) cells [3,5-10]. Although some variations have been identified (such as the less common single cell C_4_ photosynthesis), in most C_4_ leaves the BS cells occur as a layer around each leaf vein, with one or more layers of M cells surrounding each ring of BS cells [5-10]. This specialized leaf anatomy provides a structural framework that compartmentalizes the two stages of C_4_ carbon assimilation. Together these serve as a “CO_2_ pump” that concentrates CO_2_ within BS cells, where Rubisco is localized. Photosynthesis in kranz-type C_4_ leaves requires the cell-type specific expression of genes encoding certain CO_2_ assimilation enzymes, such as Rubisco in BS cells and PEPCase in M cells [5-7,11]. This two-cell compartmentalization and associated cell-type specificity in gene expression does not occur in C_3_ plants, which possess only one photosynthetic cell type, and where the initial CO_2_ fixation enzyme is ribulose-1,5-bisphosphate carboxylase (Rubisco).

In spite of the clearly defined biological parameters and advantages associated with C_4_ plants (cell-type specific expression, anatomical and metabolic modifications, increased nitrogen-use efficiency, adaptability to marginal habitats), molecular processes responsible for C_3_ versus C_4_ photosynthetic gene expression patterns have remained highly elusive for many years. Previous studies have shown that both transcriptional and post-transcriptional regulation are involved in BS or M cell-specific regulation of C_4_ genes [5-7,11,12]. While there are many trans-acting proteins known to be associated with the expression of plastidic- and nuclear-encoded photosynthetic genes at all regulatory levels in both C_3_ and C_4_ plants [5,6,12-17], most of these are not directly implicated in determining BS versus M cell-specific gene expression. Some of the few transcription factors shown to be associated with C_4_ development are members of the *Golden 2-like* (*GLK*) gene family [5,11,12]. One member of this family, *Golden*2 (*G2*) is a transcriptional regulator that functions primarily within BS cells and affects the overall development of these cells in maize leaves. A paralog of this gene, *Glk1*, is abundantly expressed in M cells, where it also regulates overall photosynthetic development. Recently, it was demonstrated that a transcription factor encoded by the *Scarecrow* (*Scr*) gene is associated with the normal development of kranz leaf anatomy, affecting the morphology and plastid content of maize leaf BS cells [18]. While each of these transcription factors has significant effects on BS or M cell development, direct regulation of C_4_ photosynthetic gene expression within their respective cell types has not been demonstrated [5,11,12,18]. In fact, to date no trans-acting factors have been directly associated with the BS versus M cell-specific regulation of any individual C_4_ gene.

As the principle enzyme of photosynthetic carbon fixation, Rubisco is central to the viability, growth, and productivity of all plants. Understanding regulatory processes responsible for the production of Rubisco specifically within the leaf BS cells of C_4_ plants, and how these processes differ from the “default” C_3_-type form, is highly significant for understanding the molecular basis of this specialized photosynthetic pathway [5,7,11,12]. Rubisco is located within the chloroplasts of all plants, and is composed of eight large (LSU, 51–58 kDa) and eight small (SSU; 12–18 kDa) subunits [7,19,20]. The *rbc*L gene encoding the LSU is transcribed and translated within the chloroplasts. The SSU, encoded by a nuclear *Rbc*S gene family, is translated on cytoplasmic ribosomes as a 20-kDa precursor that is targeted to the plastids. The *rbc*L and *Rbc*S transcripts and corresponding proteins are highly abundant and coordinately regulated; the two subunits accumulate in stoichiometric amounts within the plastids [5-7,19,20]. Rubisco gene expression in C_4_ and C_3_ plants is influenced by many factors, including light, development, cell type, photosynthetic activity, and even pathogen infection [5-7]. In addition to transcriptional control, many aspects of *rbc*L and *Rbc*S expression have been shown to be controlled through mRNA processing (degradation or stabilization of transcripts) and regulation of translation. Significantly, many studies have demonstrated that in several dicot and monocot C_4_ species, including amaranth, flaveria, cleome, and maize, post-transcriptional control plays a key role in determining the BS cell-specific expression of genes encoding both Rubisco subunits [5,7]. Post-transcriptional control of cell-type specificity for *rbc*L and *Rbc*S in C_4_ plants is very stringent; even when these genes are ectopically over-expressed in maize, Rubisco accumulation remains highly specific to BS cells [21].

Plastid- and nuclear-encoded mRNAs possess specific cis-acting sequences that mediate their post-transcriptional regulation [6,13-17,22]. Cis-acting control regions can occur within the 5′ UTR, the 3′ UTR, or even the coding region of an mRNA. For plastid-encoded mRNAs, where post-transcriptional regulation is the primary regulatory determinant, nuclear-encoded proteins usually interact specifically with 5′ or 3′ UTR sequences to regulate one or more aspects of mRNA metabolism. There are a very large number of nuclear-encoded RNA binding proteins in plastids, reflecting the very large number of complex RNA metabolic processes that occur for each of the 100 or so plastid-encoded transcripts [14,16,17]. RNA modifications can include processing of 5′ and 3′ termini, intron splicing, proofreading and editing, as well as regulation of translation and stability. Several classes of RNA binding proteins have been identified and characterized in chloroplasts, many of which are highly specific for unique sequences contained within different plastid-encoded mRNAs [14,16,17,23,24]. Among these, the most predominant are the pentatricopeptide repeat (PPR) family of RNA binding proteins, with about 450 members in higher plants [16,17,25]. One PPR protein has been shown to define 5′- processing of *rbc*L mRNA [15].

This current study contributes a new member to the list of plastid-targeted RNA binding proteins that affect gene expression in chloroplasts, in this case through its selective interaction with *rbc*L mRNA. The RBCL RNA S1-BINDING DOMAIN protein (RLSB) was isolated from chloroplasts of a C_4_ plant by affinity-purification based on its ability to bind *rbc*L mRNA *in vitro*. This protein, encoded by the *RLSB* gene, is present and highly conserved among a wide variety of plant species, contains a conserved S1 RNA binding domain, and a plastid transit sequence. We show here that RLSB affects *rbc*L gene expression within BS chloroplasts of C_4_ maize (*Zea mays*), as well in C_3_*Arabidopsis* (*Arabidopsis thaliana*) chloroplasts. Mutation or silencing of *RLSB* led to clearly observable changes in levels of *rbc*L mRNA and LSU protein accumulation, with many associated developmental effects. This is the first cell-type specific regulatory factor to be implicated in the regulation of an individual photosynthetic gene in a C_4_ plant, its accumulation correlating tightly with the BS-specific expression of the plastidic *rbc*L gene that it regulates. This strong correlation suggests that modifications of *RLSB* gene expression from the “default” C_3_ pattern to C_4_-type BS cell-specificity, and associated cell-type specific localization of *rbc*L expression, might represent one evolutionary process enabling C_4_ expression patterns in plants that utilize this specialized pathway for photosynthetic carbon assimilation.

## Results

### Isolation of an *rbc*L mRNA binding protein from chloroplasts of a C_4_ plant

**Figure 1:**
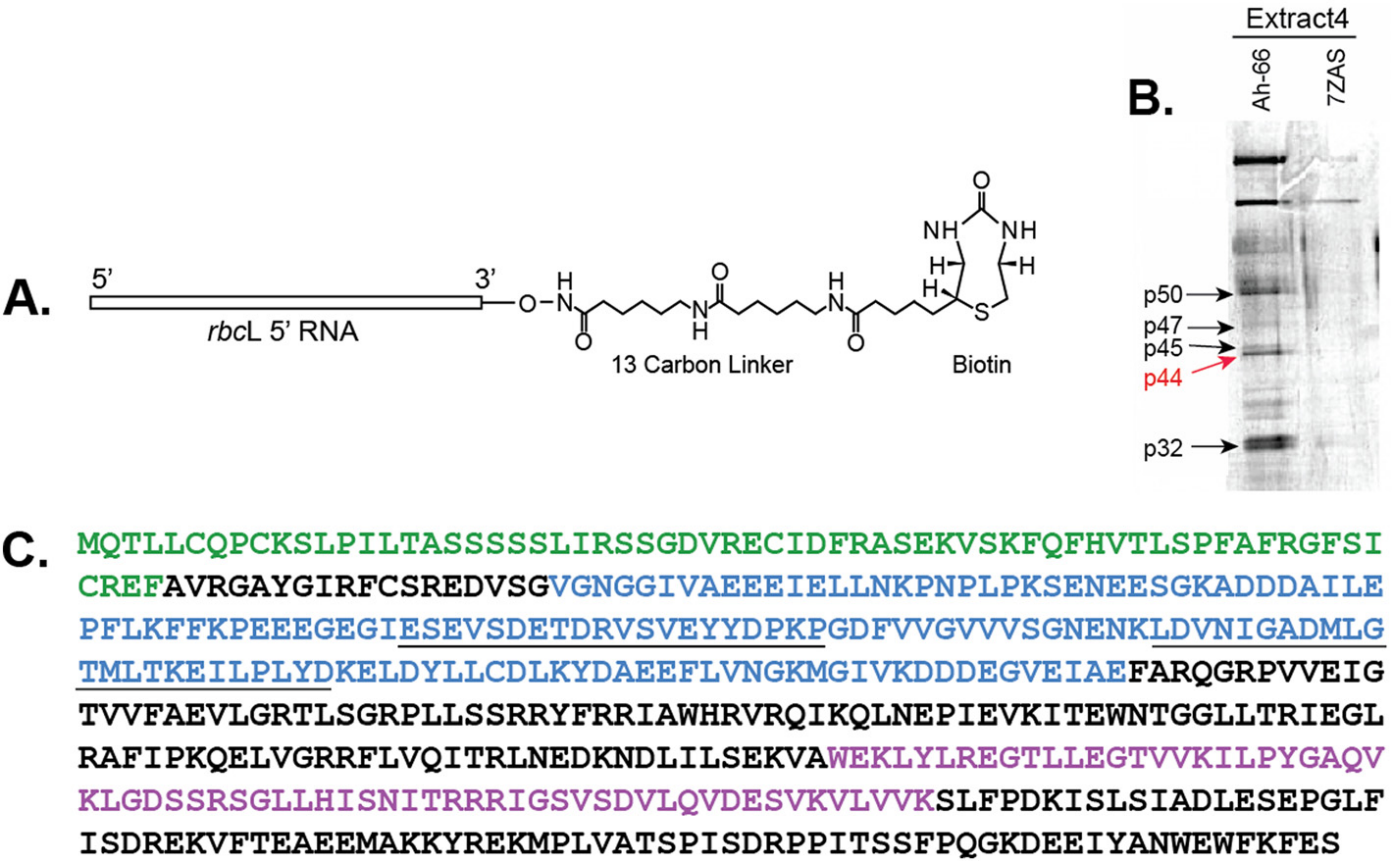
**Isolation and characterization of a plastid-targeted mRNA binding protein. A**. *rbc*L 5′ UTR probe used for affinity purification of RNA binding proteins from plastid extracts. This probe contained a 12 carbon linker and biotin at the 3′ end for attachment to streptavadin magnetic beads. **B**. Biotinylated *rbc*L and control RNAs (7ZAS, is a yeast viral UTR of similar length and AU content) incubated with plastid extracts. RNA-protein complexes were “fished” from the extracts using streptavadin magnetic beads, and then analyzed using SDS-PAGE (10% gel). Bands were excised from this gel and used for identification by Maldi-Tof/amino acid sequence analysis. Red arrow shows the position of the p44 *rbc*L mRNA binding protein (now designated RLSB). **C**. Diagram of RLSB ortholog of *Arabidopsis*. Maldi-Tof mass spectrometry/amino acid sequence analysis (Custom Biologics, Toronto, CA), and comparisons of peptide sequences with the *Arabidopsis* and other plant databases, identified one of the purified proteins (p44, red arrow in **1B**) as having properties of interest, with a plastid transit sequence and a conserved RNA binding domain. Green = plastid transit sequence (identified using (http://www.cbs.dtu.dk/services/TargetP/). Purple = conserved S1 RNA binding domain. Blue = 149 aa region expressed in *E. coli* used for affinity purification of p44 (RLSB) antibodies. The 447 nt region encoding this peptide sequence was also used for production of an RNA silencing vector in pHannibal. Underlined sequences within the blue region were used for production of peptide antibodies; the second underlined sequence (bold) also corresponds to a conserved 23 aa tryptic peptide identified in the purified amaranth protein that was identical in the *Arabidopsis* protein, and highly similar in orthologs from many other plant species (Additional file 1: Figure S1 and Additional file 2: Figure S2).

Our earlier studies identified four sites of highly specific RNA-protein interactions at the 5′ region of *rbc*L mRNA in plastid extracts from the C_4_ dicot amaranth [26]. These were found only in light-grown plants, when Rubisco synthesis occurred, and not in etiolated plants, when Rubisco synthesis did not occur. We hypothesized that RNA-protein interactions such as these might be involved in regulating BS cell-specific *rbc*L gene expression, as well as light-mediated regulation, in the leaves of C_4_ plants. Two types of RNA “bait” molecules were used for affinity purification of chloroplast proteins that specifically interact with *rbc*L 5′ RNAs [26]; an *in vitro*-transcribed RNA corresponding to *rbc*L 5′ RNA of the C_4_ dicot amaranth, a region previously shown to interact with plastidic proteins *in vivo* (beginning at the 5′ end of the processed *rbc*L mRNA at −66 and extending to +60 in the coding region), and a control 7Z-AS RNA, a yeast viral 3′ UTR of similar size and AU content [26,27]. These transcripts were biotin-tagged at the 3′ end to allow binding of the RNA to streptavidin magnetic beads (Figure 1A). The 5′ RNA-biotin-streptavidin beads were incubated with plastid extracts prepared from leaves of the C_4_ plant amaranth, using preparatory and binding conditions previously optimized for these leaves [26]. The bead-bound RNA-protein complexes were washed and isolated by magnetic separation. Figure 1B shows affinity purified plastid proteins after incubation with *rbc*L or control RNAs, separated and visualized by SDS-PAGE.

At least six distinct affinity-purified proteins were specifically captured with *rbc*L 5′ RNA, and not with the control viral RNA, ranging in size from 30–70 kDa (Figure 1B). Analysis of tryptic peptide sequences using Maldi-Tof mass spectrometry (Custom Biologics, Toronto, CA) indicated that one of the purified proteins (p44, red arrow in Figure 1B) had similarity to proteins in the database containing a plastid transit sequence and a conserved S1 RNA binding domain (Figure 1C, Additional file 1: Figure S1). Taken together, these characteristics identified p44 as a potential *rbc*L-mRNA binding protein in chloroplasts. This protein, now designated as RLSB, was selected for further analysis. The sequence and characteristics of the *Arabidopsis* ortholog are indicated in Figure 1C.

Highly similar orthologs of the RLSB protein were identified in more than 15 plant species including dicots, monocots, C_3_ and C_4_ species. This includes *Bienertia sinuspersici* (Gerald Edwards, personal communication), a dicot plant species that utilizes a unique single-cell form of C_4_ photosynthesis [10]. Comparative alignments of representative protein sequences are shown in Additional file 1: Figure S1 and Additional file 2: Figure S2. Overall similarities among the plant species examined range from 60% for maize-*Arabidopsis*, to 70% for maize-rice, and 90% for maize-sorghum. All of these proteins have putative plastid-targeting sequences. RLSB appears to be encoded from a single copy gene in all of the species examined.

### RLSB is specific to BS cells in C_4_ leaves

**Figure 2:**
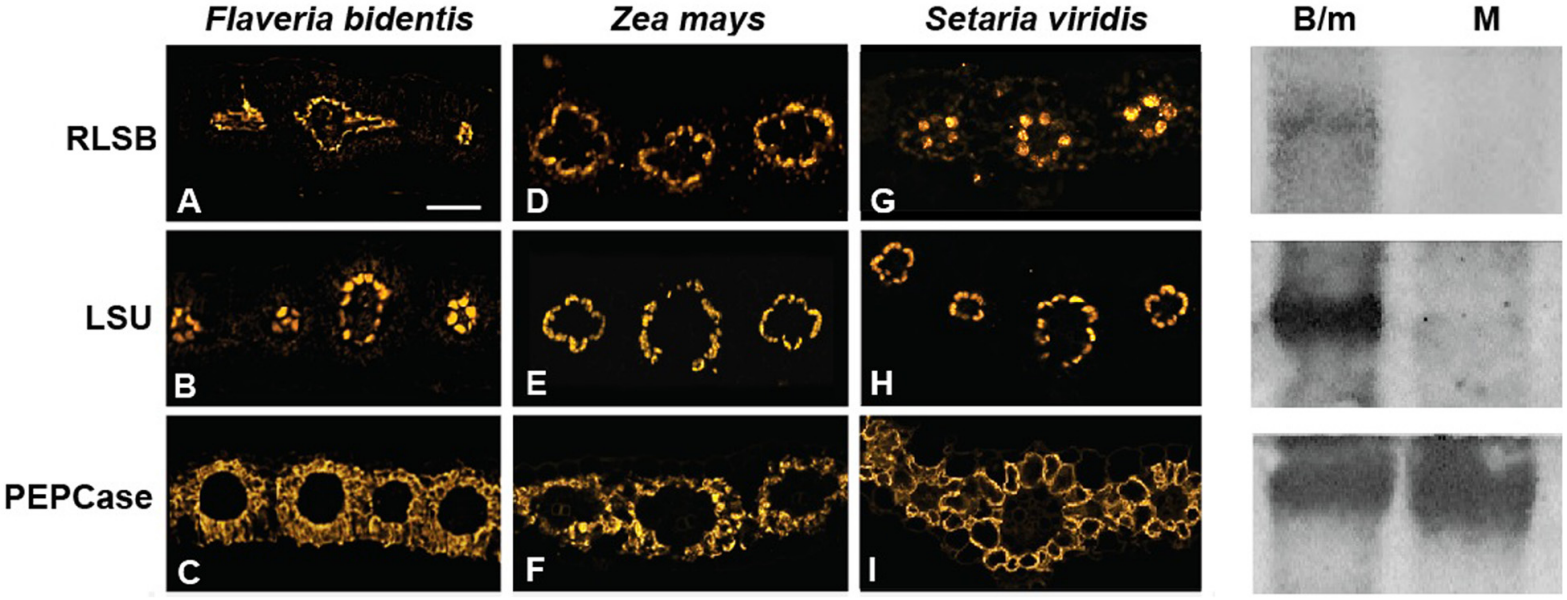
**Confocal Imaging showing co-localization of RLSB and Rubisco LSU in leaves of three C**_**4 **_**plant species.** Column 1 **(A, B, C)**: *Flaveria bidentis*, a C_4_ dicot. Column 2 **(D, E, F)**: Maize (wild type line B73), a C_4_ monocot. Column 3 **(G, H, I)**: *Setaria viridis*, a C_4_ monocot. Top row **(A, D, G)**: leaf sections from the different C_4_ species were reacted with RLSB antisera. Middle row **(B, E, H)**: leaf sections were reacted with Rubisco LSU antisera. Bottom row **(C, F, I)**: leaf sections were reacted with PEPCase antisera (as an M-specific control). Note the centripetal chloroplast positioning within bundle sheath cells of *F. bidentis*, versus the centrifugal positioning in maize and *S. viridis*. Sections treated with indicated antisera were reacted with R-phycoerythrin **(A and B)** Alexafluor 584 **(C to I)** secondary antisera and captured using the 40X objective of a Zeiss 710 LSM Confocal microscope*.* Right Panels: Immunoblot of soluble B73 maize leaf protein extracts from mechanically separated cell populations produced using the leaf rolling method. B/m, cell population enriched in BS cells, with some M cells. Equal amounts of protein were loaded into each lane. M, purified M cells. Blots were incubated with antisera against RLSB, LSU, and PEPCase.

The binding affinity of RLSB to *rbc*L mRNA in extracts from C_4_ chloroplasts presented the possibility that this binding activity might be associated with *rbc*L regulation within BS cells. If RLSB is in fact closely associated with active *rbc*L gene expression, then it would be expected that this RNA binding protein, like Rubisco, would also show specificity to BS chloroplasts in the mature leaves of Kranz-type C_4_ plant species. Immunolocalization analysis was performed with three C_4_ species shown in Figure 2; the dicot *Flaveria bidentis* (Figure 2 column 1; 3A, 3B, 3C), the monocot maize (Figure 2 column 2; 3D, 3E, 3F) and the monocot *Setaria viridis* (Figure 2 column 3, 3G, 3H, 3I). In the leaves of each of these C_4_ species, RLSB (Figure 2 top row: 3A, 3D, 3G) co-localized with Rubisco LSU (Figure 2 middle row; 3B, 3E, 3H) specifically within chloroplasts of the leaf BS cells. The sections used for Figure 2 were all taken from mature leaves (midway between the base and tip) of the indicated plant species, reacted with the primary and secondary antisera indicated, and captured using confocal imaging. Note that in *F. bidentis*, the RLSB/LSU containing chloroplasts were at the centripetal position within the BS cells (adjacent to the vascular centers), as is characteristic for this C_4_ dicot [28]. In maize and *S. viridis*, the BS chloroplasts were at the centrifugal portion (away from the vascular center), as expected for these C_4_ monocots [28]. Very low levels of 564–577 nm emission were also observed in M cell chloroplasts of sections reacted with RLSB antisera; from this imaging we cannot determine if this was due to very low levels of RLSB accumulating in these cells, or perhaps to background reactions of the affinity-purified RLSB antisera. RLSB does not appear to be an abundant protein, and sensitivity for detection needed to be increased, relative to LSU, for its fluorescent detection. As a control, the MP cell-specific enzyme PEPCase was localized specifically to the cytoplasm of leaf M cells of all three C_4_ species (Figure 2 bottom row; 3C, 3F, 3I).

To confirm that RLSB does not accumulate in the C_4_ M cells, mechanical separation of BS and M cells from wild type maize B73 was performed using the leaf rolling method [29]. This yielded a highly purified population of M cells, as well as a population that was enriched for BS cells but also contained M cells (B/m). Immunoblot analysis clearly demonstrated that RLSB, together with LSU, was present in soluble protein extracts prepared from the B/m cells, but were not detectable in extracts from the purified M cells (Figure 2, panels on the right). As a control, PEPCase was very abundant in the purified M extracts, and was also present, at slightly reduced levels, in the B/m cell extracts. It should be noted that the B/m extracts were isolated immediately after rolling out the M cells, without any additional purification steps. We have observed that RLSB degrades rapidly once the leaves have been disrupted; thus this cell population was not subjected to any further purification, leaving a significant amount of M cells remaining in the B/m extracts. The “rolled out” M cell population itself was free of contaminating BS cells, as determined by the lack of LSU in these protein extracts. These findings, together with the immunolocalization analysis, confirm that RLSB is highly specific to BS cells in the leaves of maize and other C_4_ plants.

### Specificity of RLSB binding and effects of *rlsb*-insertion mutation in the C_4_ monocot maize

Although RLSB was first identified from chloroplast extracts of the C_4_ dicot Amaranth, it’s very strong conservation across many different plant species made it feasible to employ the model C_4_ plant maize (*Zea mays*) for functional characterization. The numerous genetic and database resources available for maize allowed for mutational, developmental, and molecular analysis of RLSB in a plant with a well-defined genetic background. Some of the resources utilized for this study include those described in [30-33], as well as the Maize Photosynthetic Mutant (PML, http://pml.uoregon.edu/photosyntheticml.html), and The Plant Proteome Database (PPDB; proteomics data for the maize RLSB ortholog can be viewed at http://ppdb.tc.cornell.edu/dbsearch/gene.aspx?id=674610).

**Figure 3:**
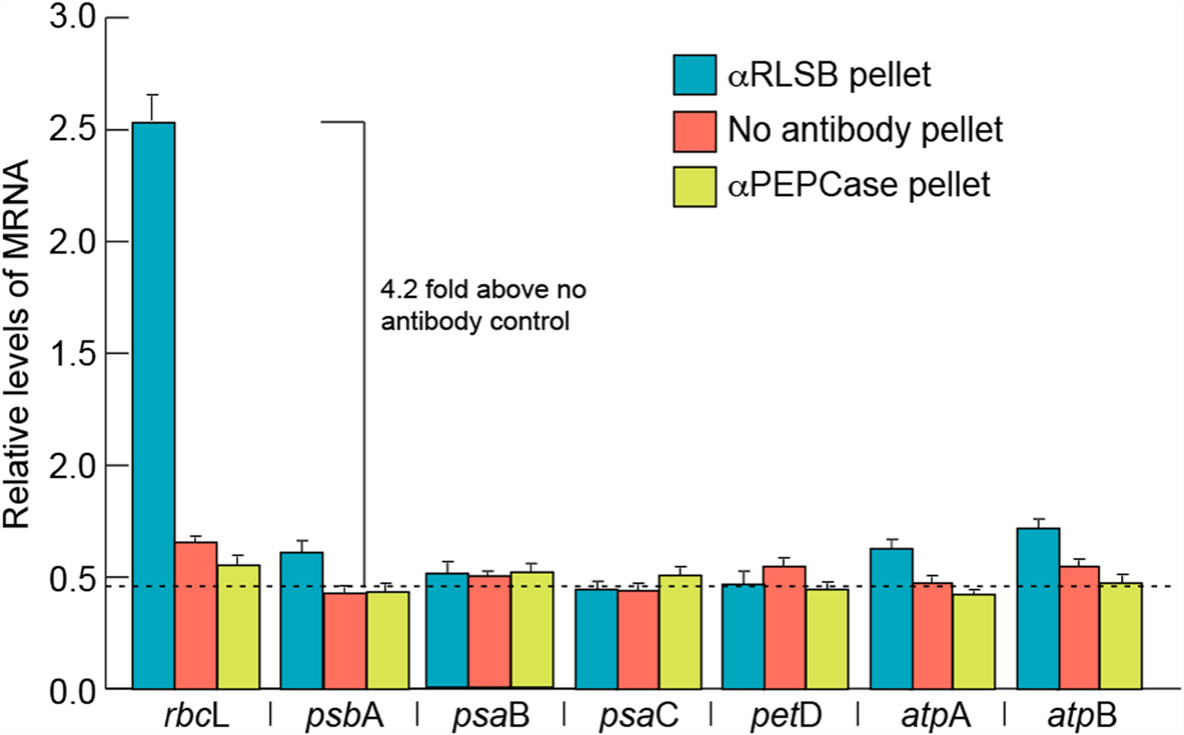
**Selectivity of RLSB binding to representative plastid-encoded transcripts of maize.** Levels of each of the indicated plastid-encoded transcripts were analyzed using RNA immunopurification and real-time quantitative PCR (RIP/qRT-PCR), as described in the text. RNA was extracted from pellet fractions following immunoprecipitations from maize chloroplasts, using antisera against RLSB, or as controls, anti-PEPCase or no added antisera. qRT-PCR was performed using primers for each of the seven transcripts indicated. All mRNA levels were quantified relative to plastid-encoded transcripts of plastid ribosomal protein (*rpl*2). Results shown are averaged from 2 repeats of 3 independent experiments. The dotted line at 0.48 indicates the average level of amplification from the no-antibody control reactions; this was used as the background value. Statistical significance was calculated using Student’s t-test. For each bar, P values were less than 0.05.

*In vitro* affinity purification of RLSB from C_4_ chloroplast extracts provided initial evidence for its selective interaction with *rbc*L mRNA. To determine if RLSB binds specifically to *rbc*L mRNA *in vivo*, we used RNA immunopurification with RLSB antisera and quantitative real-time PCR (RIP/qRT-PCR) (Figure 3). Recent studies have demonstrated the enhanced reliability and quantitative accuracy of this approach for analyzing specific protein-RNA associations, with a higher degree of enrichment, lower background, and greater dynamic range than previously used methods such as RIP-Chip (for example [34,35]). RLSB was immunoprecipated from chloroplast extracts prepared from leaves of wild type maize line B73 [32]. RNA was purified from the pellet fractions, and qRT-PCR was performed using primers for *rbc*L and, for comparison, the representative plastid-encoded transcripts *psa*B, *psb*A, *pet*D, *psa*C, *atp*A, and *atp*B, as indicated in Figure 3. As controls, RIP/qRT-PCR reactions were performed using antisera against cytoplasmic PEPCase, and with no added antisera. All of the qRT-PCR reactions were standardized relative to plastid-encoded *rpl*2 mRNA (encodes ribosomal protein Rpl2).

Strong selective association of *rbc*L mRNA with RLSB was observed in the maize chloroplast extracts (Figure 3). Of the seven plastid-encoded mRNAs examined, only sequences corresponding to *rbc*L mRNA showed high levels of amplification from the anti-RLSB pellet fraction (4.2 fold above background, as determined from the no-added antibody control reactions). Three plastid-encoded mRNAs (*psa*B, *pet*D, *psa*C) showed no amplification above background from the immunopurified RLSB pellets. Three other plastid-encoded transcripts (*psb*A, *atp*A, and *atp*B) showed only very slight levels of amplification (0.2 – 0.3 fold above the averaged background value). None of these sequences, including *rbc*L, were amplified from control PEPCase immunopurifications, or when no antisera was used (Figure 3). It is clear from this analysis that *rbc*L mRNA showed significantly greater interaction with RLSB than any of the other representative plastidic mRNAs tested. These findings confirm that the plastid-localized RLSB protein does in fact bind to plastid-encoded *rbc*L mRNA *in vivo*, with significant specificity for *rbc*L mRNA in wild type maize plastids.

The biological significance of RLSB interactions with *rbc*L mRNA in maize was investigated by making use of Mu transposon insertion mutations within the maize genomic *RLSB* ortholog. The genomic sequence of the maize *RLSB* ortholog was initially identified (within maize Genomic BAC AC211368.4) using a cDNA sequence accession #BT035293.1 (partial sequence; the full length cDNA is accession #JX650053, Additional file 1: Figure S1 and Additional file 2: Figure S2). This gene (GRMZM2G087628) is approximately 3540 nucleotides in length, with seven introns (Additional file 3: Figure S3). Mu insertions into this gene were identified by screening the maize Photosynthetic Mutant Library (PML) at the University of Oregon (http://pml.uoregon.edu/photosyntheticml.html), using primer sets specific for *RLSB* and the Mu transposon borders (see Methods). Two independent lines were isolated and designated as *rlsb-1* and *rlsb-2*. Genomic mapping from both ends of the Mu transposon indicates that each line contains a single insert within the *RLSB* gene, located within the first exon; these occur at positions just 37 nt apart. The insertion in *rlsb-1* is nearly adjacent to the 5′ splice site of intron 1, positioned 8 nt upstream of the first 5′ intron junction. The insertion in *rlsb-2* is positioned 45 nucleotides upstream of the first splice junction (red stars in Additional file 3: Figure S3). Both Mu insertions occur within a protein coding exon, and would affect the mature RLSB protein within its N-terminal portion, just after the predicted cleavage site for the plastid transit sequence. When homozygous, the phenotype of each line is identical; the leaves start out as virescent-yellow, and gradually begin to green from the tip to the base as they grow and develop (Additional file 4: Figure S4, Top panels). The mutants grow more slowly than wild type, so that leaf development is delayed approximately one day. Genetic crosses demonstrate that the two mutants do not complement; most of the experiments presented here were done using *rlsb-1*/*rlsb-2* double mutants.

An analysis of these maize insertion mutants must be undertaken within the framework of the maize leaf developmental gradient. A maize leaf originates and grows outward primarily from an intercalary meristem located at the base of the leaf [36,37]. This leads to the development of a linear gradient of cells occurring along the entire length of a growing maize leaf, with younger cells occurring at the lower (basal) regions, and older cells at the outer (towards the tip) regions. Rubisco mRNA and protein accumulation increase along this maize leaf gradient, with the transcripts appearing slightly ahead of their corresponding proteins [5,7]. In illuminated maize leaves, Rubisco mRNAs and subunit proteins are specifically localized to BS precursors at their first occurrence, and remain specific to BS cells across the entire developmental gradient.

**Figure 4:**
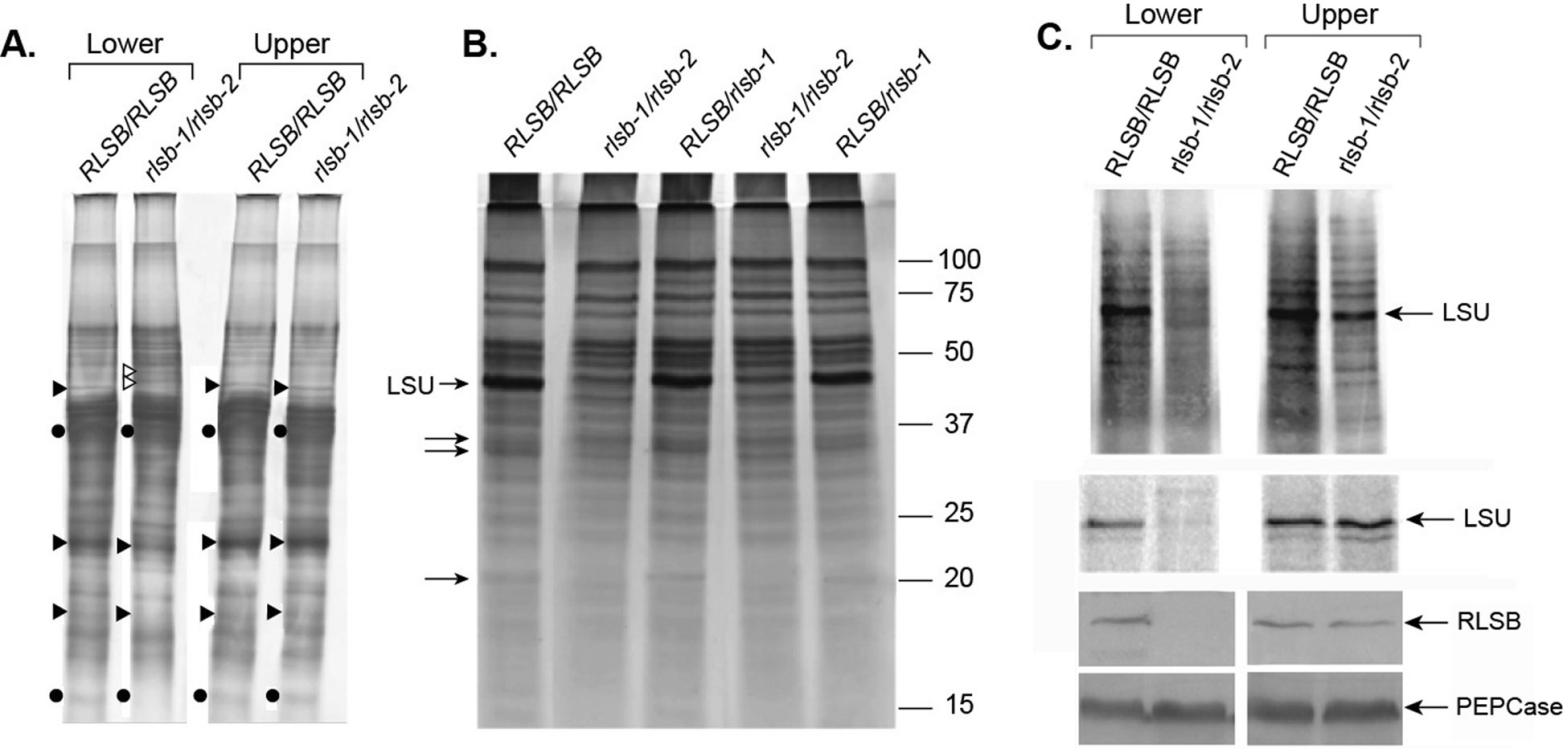
**Protein accumulation and synthesis in *RLSB/RLSB *and *rlsb-1/rlsb-2 *double mutant sibling plants.** For each panel, proteins were isolated from lower (lower third) or upper (upper third) regions of the first or second emerging leaves (4 – 6 cm in length) from each genotype. Panel **A**: Equalized amounts of total proteins (soluble plus membrane) isolated from these regions were loaded and separated by SDS-PAGE, and silver stained. The positions of LSU and SSU (solid circles), unidentified proteins reduced in mutant leaf base (solid triangles), and unidentified proteins increased in mutant leaf base (open triangles) are indicated. Panel **B**: Soluble protein accumulation in lower leaf regions of *RLSB/RLSB*, heterozygote *RLSB*/*rlsb-1*, and mutant maize *rlsb-1/rlsb-2* plants. Positions of the Rubisco LSU, as well as three other soluble proteins affected in the mutant, are indicated (arrows). Note that in this panel, the LSU band is overlapping (and slightly below) another unidentified protein, which somewhat obscures its reduction in the double mutant plants. Panel **C**, top: *In vivo* synthesis of total soluble proteins in lower and upper leaf regions. Protein extracts were prepared from leaf disks labeled with ^35^S-met/cys for one hour, as described in Methods. Equalized amounts of labeled extract were separated by SDS-PAGE and visualized using a phosphorimager. Position of the LSU is indicated. Panel **C** middle: Immunoprecipitation of LSU from the ^35^S-labeled extracts. LSU protein was immunoprecipitated from equalized amounts of labeled plant extract, separated by SDS-PAGE, visualized and quantitated using a phosphorimager. Panel **C**, bottom: Immunoblot showing accumulation of RLSB and PEPCase (as a loading control) proteins in lower and upper regions of *RLSB/RLSB* and *rlsb-1/rlsb-2* maize leaves. Equalized amounts of total (soluble plus membrane) proteins from *RLSB/RLSB* and *rlsb-1/rlsb-2* leaves were separated by SDS-PAGE and analyzed by immunostaining using the antisera indicted, as described in Methods.

The phenotype of these *RLSB* insertion mutants indicates that *rlsb-1* and *rlsb-2* affect early photosynthetic development, as observed in lower regions of the maize leaf (Additional file 4: Figure S4, Top Panels). An overview of total protein accumulation (soluble plus membrane) demonstrated that the overall protein profiles were mostly similar for both *RLSB* insertion mutant (*rlsb-1*/*rlsb-2*) and non-insertion mutant (*RLSB/RLSB*) leaves in the lower leaf regions (lower 1/3 of the leaf, earlier developmental stage), although there were clearly differences in levels for a few proteins bands (Figure 4A). Some of these were decreased in lower regions of the mutant leaves, while others were increased (indicated in Figure 4A). Most notably, protein bands migrating at the position of the Rubisco LSU and SSU were significantly reduced in lower regions of the *rlsb-1*/*rlsb-2* leaves, relative to *RLSB/RLSB*. At the leaf upper regions (upper 1/4 of the leaf, more advanced developmental stage), levels of the Rubisco protein bands were elevated, so that identical amounts were present in this portion of the mutant and non-mutant leaves. Similarly, in *rlsb-1*/*rlsb-2* leaves, protein bands that were altered in lower regions were present at normal *RLSB/RLSB* levels in the upper regions. Thus, at this level of analysis, insertion mutants of *RSLB* had different effects on protein accumulation in the lower versus the upper portion of the maize leaf.

Reductions in Rubisco LSU levels in lower regions of the *rlsb-1*/*rlsb-2* leaves were more clearly discernible when soluble proteins were analyzed separately (Figure 4B). As with total proteins (Figure 4A), soluble protein profiles were mostly similar for *rlsb-1/rlsb-2* mutant and *RLSB/RLSB* leaves. We detected at least 3 prominent soluble proteins, in addition to the dramatically reduced LSU, that were clearly reduced in lower leaf regions of the mutant plants. Several differences in protein composition were also observed for membrane-bound proteins in the lower leaf regions of mutant plants (not shown). These differences in accumulation of the LSU and other proteins between *RLSB* mutant and non-mutant plants were not observed at the leaf upper regions (not shown). In plants heterozygous for the *rlsb* mutation (*RLSB/rlsb-1*, *RLSB/rlsb-2*), accumulation of LSU (and the other indicated proteins) was not affected in either leaf region; the protein profiles for these plants were identical to *RLSB/RLSB* (Figure 4B), demonstrating that the insertion mutant is recessive.

Reductions in LSU protein accumulation were accompanied by reduced *in vivo* synthesis of the LSU protein in the lower region of the mutant leaves, but not in the upper region (Figure 4C, top and middle panels). Incubation of leaf disks from *rlsb-1/rlsb-2* and *RSLB/RSLB* plants with ^35^S-met/cys labeling solution for one hour, followed by isolation of soluble proteins and separation of equalized protein samples by SDS-PAGE, showed greatly reduced *in vivo* synthesis of the LSU protein in lower regions of the mutant leaves (Figure 4C, top panel). While significant differences in synthesis were found for the LSU protein in the lower regions, the majority of proteins observable in the labeled extracts showed no differences between *rlsb-1*/*rlsb-2* and *RLSB/RLSB*. This mostly selective reduction in LSU synthesis was not observed in the upper regions. Immunoprecipitation of LSU protein confirmed that LSU synthesis was significantly reduced in the lower regions, but not the upper regions, of the *rlsb-1/rlsb-2* mutant maize leaves (Figure 4C, middle panel). As demonstrated by immunoblot analysis using RLSB antisera (Figure 4C, bottom panels), greatly reduced levels of *in vivo* LSU synthesis in lower regions of the *rlsb-1/rlsb-2* leaves correlated very closely with reduced levels of RLSB protein accumulation at these same regions. Comparatively, the upper regions of the *rlsb-1/rlsb-2* leaves, and both regions of RLSB/RLSB leaves, all showed much higher levels of RLSB protein accumulation, corresponding with their higher levels of LSU synthesis.

The combined data of Figure 4 indicate that the accumulation and synthesis of Rubisco LSU protein was delayed, and not completely eliminated, in the *rlsb-1/rlsb-2* maize leaves. The loss or reduction of the RLSB mRNA binding protein was accompanied by reduced production of the Rubisco LSU protein, as well as the other observable effects, only during early leaf development in the lower leaf region. As developmental age advanced along the maize leaf gradient, the effects of this mutation appear to be attenuated, so that in the more developmentally advanced outer leaf regions, LSU synthesis and accumulation reached normal levels.

**Figure 5:**
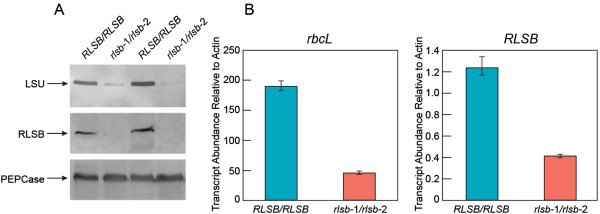
**Accumulation of Rubisco LSU and RLSB protein and mRNA in lower leaf regions from *RLSB/RLSB *and *rlsb-1/rlsb-2 *maize seedlings.** Panel **A**: Immunoblot showing accumulation of LSU, RLSB, and PEPCase (as a loading control) proteins in lower regions (lower third) of *RLSB/RLSB* and *rlsb-1/rlsb-2* maize seedlings, using the first or second emerging leaves (4 – 6 cm long). Equalized amounts of total (soluble plus membrane) proteins from non-mutant *RLSB/RLSB* and mutant *rlsb-1/rlsb-2* leaves were separated by SDS-PAGE, and subjected to immunoblot analysis as described for Figure 4, using the indicated antibodies. Panel **B**: Real-time quantitative PCR showing relative levels of mRNA accumulation for *rbc*L and *RLSB* transcripts in *RLSB/RLSB* and *rlsb-1/rlsb-2* maize seedlings. Quantification of transcript levels was standardized to actin mRNA. Data is averaged for four wild type and four mutant siblings, with three repeats run for each of the plant samples. Note differences in scale for panels showing *rbc*L and *RLSB* mRNAs, indicating that these two transcripts accumulate to substantially different levels in both wild type and mutant plants. Statistical significance was calculated using Student’s t-test. For each bar, P values were less than 0.05.

Immunoblot analysis confirmed the reduced accumulation of both Rubisco LSU protein and RLSB proteins in lower regions of the *rlsb-1*/*rlsb-2* maize leaves. Relative to the same region of *RLSB/RLSB* leaves, LSU was reduced approximately 12–15 fold in the *rlsb-1*/*rlsb-2* plants (Figure 5A, top panel, based on phosphorimager software analysis). RLSB was not detectable in the *rlsb-1*/*rlsb-2* leaf lower regions using these same conditions (Figure 5A, middle panel). A digitally enhanced image of the middle panel of Figure 5A demonstrates that RLSB did in fact accumulate in the lower leaf regions of *rlsb-1*/*rlsb-2* mutants, but at greatly reduced levels (Additional file 5: Figure S5, top panel). In fact, this longer exposure reveals that one of the *rlsb-1/rlsb-2* mutants had slightly higher levels of RLSB, relative to a different *rlsb-1/rlsb-2* mutant (compare the second and fourth lanes of Additional file 5: Figure S5, top panel). This was correlated closely with slightly higher levels of LSU for this same plant (compare the second and fourth lanes of Figure 5A, top panel).

Analysis of mRNA levels using qRT-PCR indicated in *RLSB/RLSB* maize leaves, *rbc*L mRNA was present at much higher levels than *RSLB* mRNA (Figure 5B). In fact, *rbc*L transcripts were more than 150-fold more abundant (note the difference Y-axis scales in Figure 5B). This difference in relative levels of the two mRNAs may not reflect respective levels of protein accumulation in the *RLSB/RLSB* maize leaves, since both RLSB and LSU were easily detected with similar exposure levels of the immunoblots. In lower portions of the *rlsb-1*/*rlsb-2* mutant maize leaves, both *rbc*L and *RLSB* transcript levels were correspondingly reduced (3.5-4.5 fold, respectively) relative to the same region of *RLSB/RLSB* leaves (Figure 5B). Thus, insertion mutagenesis of *RLSB* reduced but did not completely eliminate *RLSB* and *rbc*L expression, allowing for low but still detectable levels of both mRNAs to accumulate in lower *rlsb-1*/*rlsb-2* leaf regions. The fact that mRNA levels for both transcripts were reduced in approximate coordination further supports a regulatory connection between RLSB and levels of *rbc*L gene expression, involving regulation at the level of *rbc*L mRNA accumulation. Furthermore, it is notable that the reduced levels of *RLSB* and *rbc*L transcripts in the lower leaf regions of *rlsb-1/rlsb-2* plants were not reflective of actual RLSB and LSU protein accumulation. Most significantly, LSU protein levels were reduced more dramatically than *rbc*L mRNA in lower regions of the *rlsb-1*/*rlsb-2* mutant leaves (12–15 fold versus approximately 4-fold, respectively), suggesting that utilization of the *rbc*L mRNA for translation was also affected by reduced RLSB.

**Figure 6:**
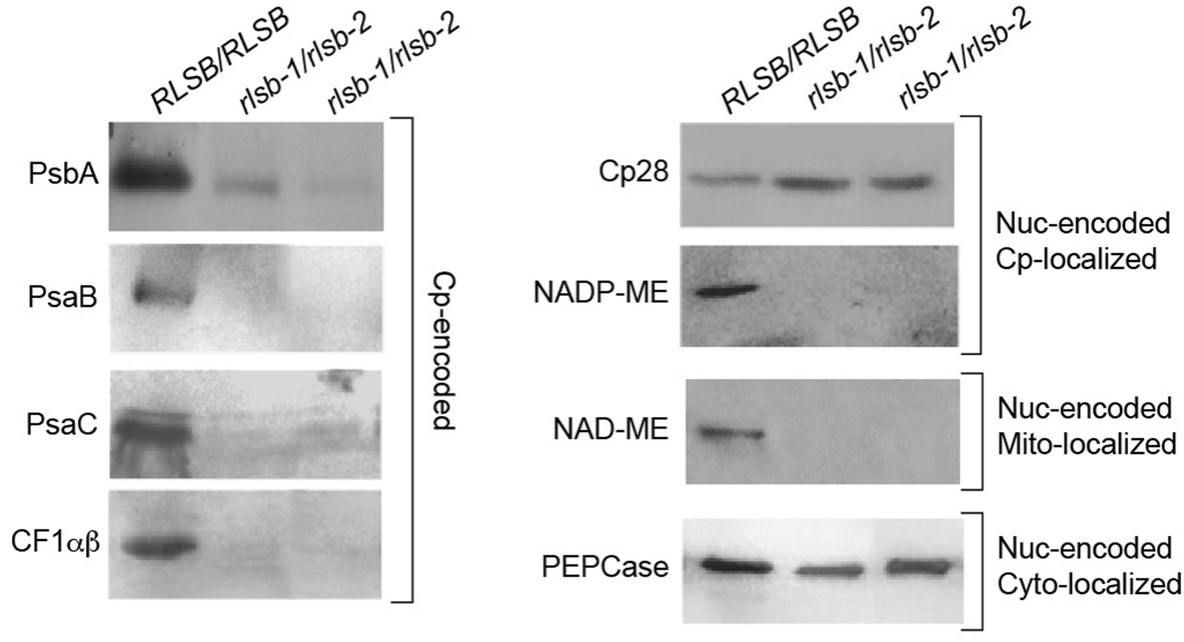
**Accumulation of various plastid- and nuclear-encoded proteins in leaf basal regions of *RLSB/RLSB *and insertion mutant *rlsb-1/rlsb-2 *maize seedlings.** Equalized amounts of total (soluble plus membrane) proteins from non-mutant *RLSB/RLSB* and mutant *rlsb-1/rlsb-2* leaves were separated by SDS-PAGE, and subjected to immunoblot analysis as described for Figure 4, using the indicated antibodies. Cp-encoded, chloroplast-encoded proteins. Note that CF1αβ antisera reacts to both the alpha and beta subunits, which run at the same position on this gel. Nuc-encoded Cp-localized, nuclear-encoded proteins targeted to the chloroplast. Nuc-encoded Mt-localized, nuclear-encoded protein targeted to the mitochondria. Nuc-encoded Cyto-localized, nuclear-encoded protein localized within the cytoplasm.

While RLSB shows strong selectivity in binding to *rbc*L mRNA, the effects of the *rlsb-1/rlsb-2* mutation extend beyond this single plastid gene. In addition to LSU, lower leaf regions of the double mutants showed significant reductions in the accumulation of several representative plastid- and nuclear-encoded proteins (Figure 6). Also similar to LSU, the reductions described below occurred only in the lower leaf regions of *rlsb-1/rlsb-2* plants; in the upper leaf regions, levels had recovered to those of *RLSB/RLSB* leaves (data not shown). Decreased RLSB in lower regions of the *rlsb-1*/*rlsb-2* leaves was associated with reductions of five other representative plastid-encoded proteins (Figure 6, left panel). PsaB, PsaC, PsbA, and CF1αβ all showed *rlsb-1*/*rlsb-2* mutation-associated reductions that were similar to or greater than LSU (compare Figures 5A and 6). Although there was a trend for some chloroplast-encoded transcripts to have reduced accumulation in the *rlsb-*1/*rlsb-*2 lower leaf regions (Additional file 6: Figure S6A), these reductions were not as dramatic as the protein reductions observed. The most significant reduction occurred for *pet*D (three-four fold); reductions in *psa*B, *psa*C, and *atp*B, while statistically significant, were less than two fold. Transcripts for *psb*A, *atp*A and *rpl*2 showed no significant reductions. Thus, within the chloroplast itself, reductions in RLSB levels were accompanied by consistent drop in levels of many photosynthetic proteins, with variable reductions in levels of different plastid-encoded mRNAs. Plastid-encoded ribosomal RNAs also showed a slight, but not statistically significant, reduction in accumulation in the affected *rlsb-*1/*rlsb-*2 leaf regions (Additional file 6: Figures S6B and S6C). Severe reductions in protein accumulation such as these, together with moderate reductions in the accumulation of some mRNAs, might be consistent with a more global effect on plastic translation, as described for maize ribosome assembly mutants [38]. However, the lack of any significant effect on ribosomal rRNAs, as would occur be in the case of a general translation mutation, makes it highly unlikely that RLSB would be a member of this class of basic translational regulators. In addition, it is important to consider that, while RLSB is specific to BS cells, all of the other plastid-encoded proteins that were affected would normally accumulate in both BS and M cells, with the photosystem II-associated PsbA protein in fact being most abundant in M plastids [5,11,39]. It therefore also highly unlikely that the reduced levels of PsbA, PsaB, PsaC, and CFαβ in the *rlsb-*1/*rlsb-*2 lower leaf regions could be a direct result of reduced RLSB accumulation, since this protein is not present in M cell chloroplasts in any case.

Most interestingly, the *rlsb-1/rlsb-2* mutation affected two representative nuclear-encoded proteins as well. The C_4_ photosynthetic NADP-ME-dependent malic enzyme (NADP-ME, nuclear-encoded, plastid targeted) was significantly reduced (below detectable levels). Similarly, the non-C_4_ NAD-dependent malic enzyme (NAD-ME, nuclear encoded, targeted to mitochondria) was also greatly reduced (below detectable levels) in lower regions of the *rlsb-1*/*rlsb-2* mutants (Figure 6). In contrast, levels of an RNA binding protein known as CP28 (nuclear-encoded plastid targeted protein [16,40] were not at all affected in the *rlsb-1*/*rlsb-2* and *RLSB/RLSB* plants. Similarly, the C_4_ photosynthetic PEPCase (nuclear encoded, cytoplasmic) was not affected in any region of the *rlsb-1*/*rlsb-2* leaves. The contrasting effects of the *rlsb-1/rlsb-2* mutation on two different nuclear-encoded plastid targeted proteins, Cp28 and NADP-ME, is particularly striking. If this were a direct result of reduced RLSB, or an indirect process inhibiting their import/accumulation due to reduced chloroplast function, then both proteins should have been similarly impacted. Similarly, reductions in a plastidic RNA binding protein would not be expected to directly affect the accumulation of a metabolic protein targeted to the mitochondria.

The multiple levels of analysis presented here provide strong evidence that in maize leaves, reduced accumulation of the *rbc*L mRNA binding protein RLSB leads to corresponding reductions in *rbc*L mRNA accumulation and LSU synthesis within BS chloroplasts. These findings support a direct effect on post-transcriptional *rbc*L gene expression, with an impact on both mRNA stability and translation. Indirect effects resulting from reduced RLSB and *rbc*L expression in the double mutants extend to proteins that are encoded, synthesized, and accumulate within other cell types and other cell compartments.

### RLSB localization and basic “default” function in C_3_ plants

RLSB is highly conserved across a broad range of C_4_ as well as C_3_ plant species (Additional file 1: Figure S1, Additional file 2: Figure S2). In consideration of this very strong conservation, we hypothesized that RLSB shares a common *rbc*L regulatory role in all plants, and may have been recruited from a more basic role in C_3_ species (“default” *rbc*L regulatory patterns) to function as a cell-specificity determinant in C_4_ plants (more specialized C_4_*rbc*L regulatory patterns). *Arabidopsis*, with its extensive genetic, molecular biology, and genomic resources ([41], http://www.arabidopsis.org), provides an ideal model system for comparative RLSB functional analysis in a plant that utilizes the C_3_ pathway of CO_2_ assimilation [5-7].

**Figure 7:**
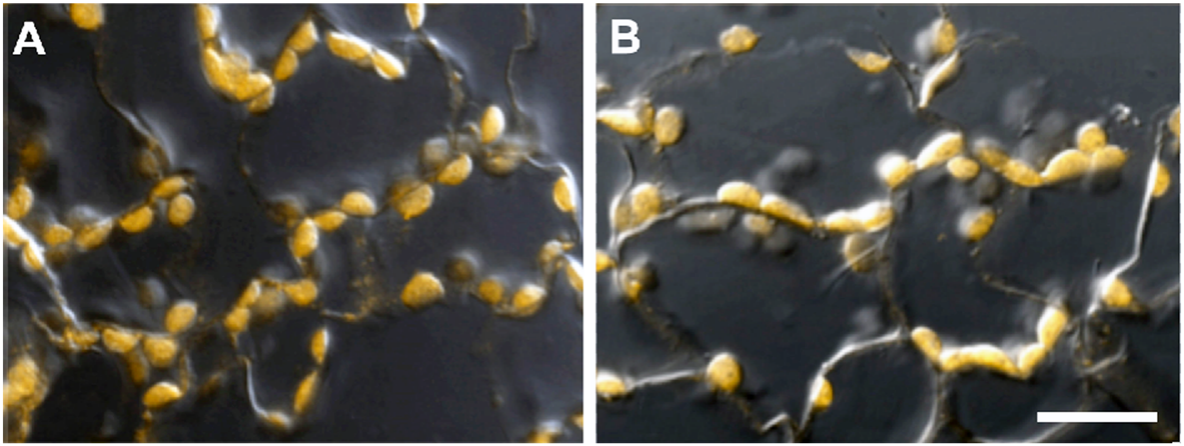
**Immunolocalization of RLSB and Rubisco LSU proteins in leaf sections of the C**_**3 **_**plant *Arabidopsis*.** Panel **A**: Confocal/DICI image of *Arabidopsis* leaf section reacted with RLSB primary antiserum. Panel **B**: Confocal/DICI image of *Arabidopsis* leaf section reacted with LSU primary antiserum. *Arabidopsis* leaf sections were incubated with the indicated primary antiserum, and then Alexafluor 546 conjugated secondary antibody. Images were captured using the 40X objective of a LSM 710 “in tune” confocal microscope. Fluorescent immunolocalization was combined with bright field DICI to clearly show plastid localization for both proteins. bar = 20 μM.

Photosynthesis in C_3_ plants occurs primarily within leaf mesophyll cells. This general classification of photosynthetic cells makes up the interior of the leaf (between the upper and lower epidermis, excluding vascular cells) and includes the palisade and spongy parenchyma [42,43]. In leaf sections of *Arabidopsis*, confocal fluorescent imaging, superimposed on a DIC image, clearly establishes the co-localization of RLSB and LSU proteins within mesophyll cell chloroplasts (Figure 7). A lower magnification overview of similar immunolocalizations indicted that both chloroplast proteins were distributed throughout *Arabidopsis* leaf mesophyll cell population, with no cell-type preferential or distributional accumulation patterns detected within this population (Additional file 7: Figure S7).

**Figure 8:**
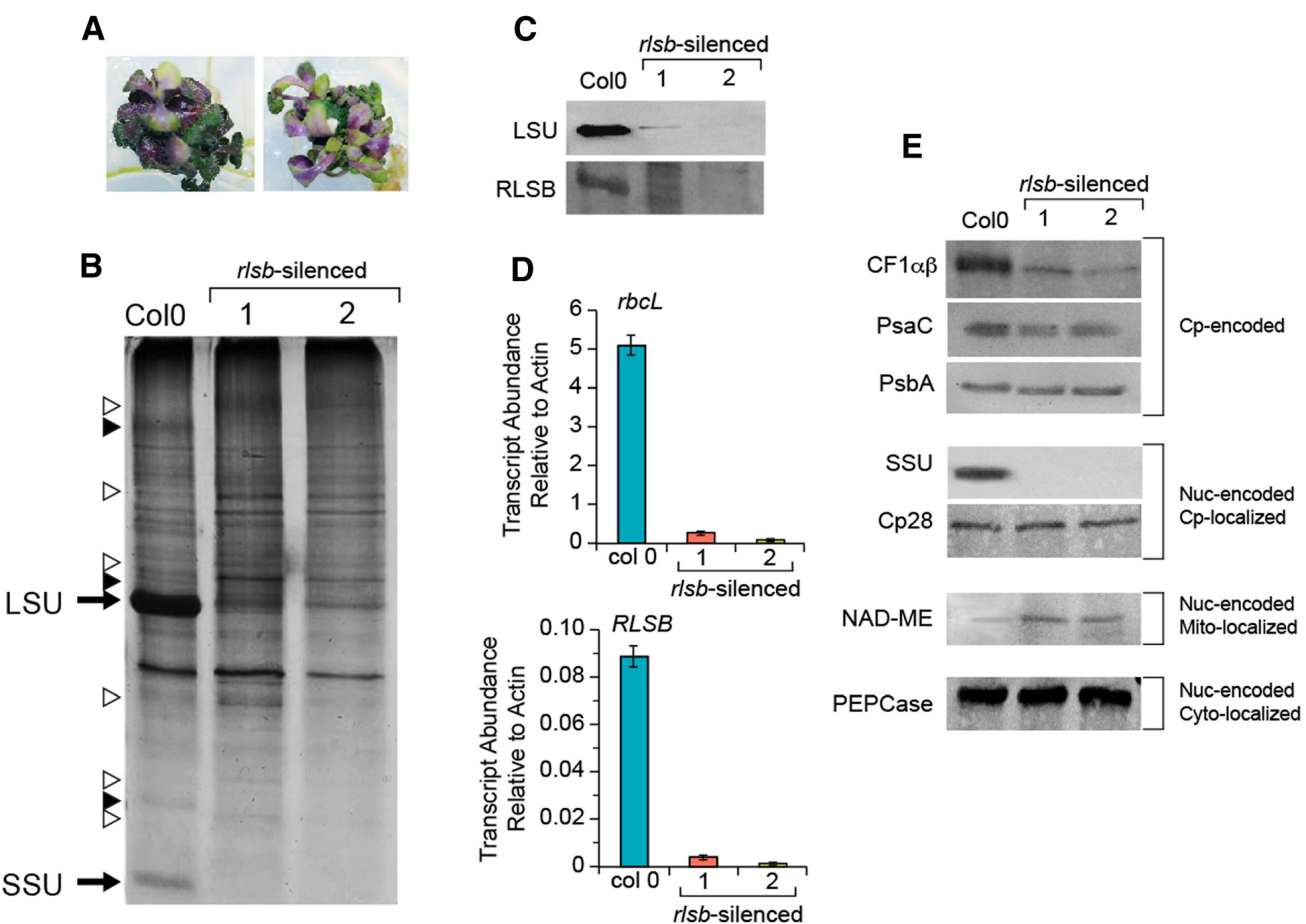
**RNA silencing of *RLSB *in the C**_**3 **_**plant *Arabidopsis*.** Panel **A**: Morphology of two *rlsb-*silenced plants. 12-week-old *rlsb*-silenced *Arabidopsis* grown on MS media supplemented with 8% sucrose. Panel **B**: Total soluble proteins isolated from leaves of wild type (Col0) and two representative *rlsb-*silenced (1 and 2) *Arabidopsis* plants were separated by SDS-PAGE and silver stained. The positions of LSU and SSU are indicated. Unidentified proteins that were reduced or increased in the silenced plants relative to wild type (open or solid triangles, respectively) are indicated. Panel **C**: Western blot showing levels of accumulation for LSU and RLSB proteins in leaves from Col0 and two *rlsb-*silenced *Arabidopsis* plants. Total maize leaf proteins were separated by SDS-PAGE and analyzed by immunostaining as described in Methods. Panel **D**: Real-time quantitative PCR showing relative levels of mRNA accumulation for *rbc*L and *RLSB* in leaves from Col0 and the two *rlsb-*silenced *Arabidopsis* plants. Quantification of transcript levels was standardized to actin mRNA. Data is averaged for four wild type and four mutant siblings, with three repeats run for each of the plant samples. Note differences in scale for panels showing *rbc*L and *RLSB* mRNAs. Panel **E**: Western blots showing accumulation levels of additional proteins in Col0 and the two *rlsb-*silenced *Arabidopsis* plants. Protein extracts used for panel **C** were separated by SDS-PAGE and detected with the indicated antibodies as described*.* Cp-encoded, chloroplast-encoded proteins (note that CF1 alpha and beta subunits run at the same position on this gel), Nuc-encoded Cp-localized, nuclear-encoded proteins targeted to the chloroplast. Nuc-encoded Mt-localized, nuclear-encoded proteins targeted to the mitochondria. Nuc-encoded Cyto-localized, nuclear-encoded proteins localized within the cytoplasm. For all of the protein and RNA samples analyzed in this Figure, equalization for isolation, loading, and analysis was based on using equalized wet weights of starting leaf material.

To determine if RLSB is associated with *rbc*L expression in a C_3_ plant, a 447 bp inverted repeat fragment of the *Arabidopsis RLSB* ortholog was expressed and used to induce *RLSB* silencing in *Arabidopsis*. Seed from floral-dipped plants were germinated and grown on MS media containing Kanamycin with 3% sucrose. To maintain viability, Kanamycin-resistant plants, which showed very slow growth, were transferred two weeks after germination to MS media containing 8% sucrose without further Kanamycin selection. Six confirmed *rlsb-*silenced plants were selected for further analysis; all produced nearly identical data. The data sets shown in Figure 8 were obtained from two of these plants that were found by initial protein analysis to have either the least (*rlsb*-silenced 1) or most severe (*rlsb*-silenced 2) reductions in LSU accumulation among the six.

Plants expressing the *rlsb-*silencing construct were easily discernable by their altered morphologies. These included severely reduced shoot growth, altered shoot morphology (there was no typical leaf rosette organization), and purple coloration of the leaves (Figure 8A). The silenced plants did not survive after transfer to soil, and were maintained on the high-sucrose media continuously. The silenced plants rarely produced bolts or flowers, and did not survive longer than 30 days. Seed from control (non-transformed) Col0 plants, germinated and grown using the same 3% and 8% sucrose-media and growth conditions, but without the initial Kanamycin selection, did not show any of these silencing phenotypes (Additional file 4: Figure S4, bottom panels). The size of the *rlsb*-silenced plants varied between the lines, showing size reductions in overall shoot growth of approximately one-third to one-fourth by 6 to 8 weeks after germination, when compared to Col0 grown on the same media under the same sterile conditions. Root growth on the transgenic plants was also impeded, but to a lesser extent.

Observations of total soluble protein accumulation revealed striking reductions in levels of protein bands corresponding to the Rubisco LSU and SSU in the *rlsb-*silenced plants (Figure 8B). There were also a few easily observable changes in several unidentified proteins that either increased or decreased in the silenced plants, relative to the controls (Figure 8B). Aside from these, overall patterns of protein accumulation were mostly similar in silenced and wild type Col0 plants. To better understand the levels, range, and specificity of proteins affected by silencing of *RLSB*, immunoblot analysis was used to check for any possible changes in a range of representative proteins.

Using antisera against LSU and RLSB, dramatic and corresponding reductions in the accumulation both proteins were observed in the *rlsb*-silenced plants, relative to wild type Col0 (Figure 8C, first and second panels). Levels of LSU protein accumulation varied somewhat between different silenced plants (compare the second and third lanes of Panel 7C), but were always considerably lower than in wild type (25–50 fold for *rlsb*-silenced 1 and 2, respectively, based on digital imaging analysis). Longer exposures of the immunoblot in the top panel showed that very low levels of LSU protein did in fact accumulate in the silenced plants (Additional file 5: Figure S5, bottom panel). Although RLSB protein was not detected in any of the silenced plants, very low levels of accumulation cannot be ruled out. Detection of this protein by immunoblot required longer exposures even in wild type *Arabidopsis* (the blot in the second panel of Figure 8C required longer exposure time than the other panels), possibly due to lower steady-state levels of accumulation/stability, or reduced sensitivity of RLSB antibody, relative to LSU.

Analysis of mRNA levels using qRT-PCR indicated that in wild type Col0 plants, *rbc*L mRNA was considerably more abundant than *RLSB* mRNA (55-fold more abundant, note the difference in y-axis scales in Figure 8D). Although such dramatic differences in transcript levels may not be reflective of final protein accumulation, the data of Figure 8C and 8D do indicate that the RLSB may be produced or accumulate at lower levels than LSU in plastids of wild type *Arabidopsis*. In comparing changes in relative levels of *rbc*L and *RLSB* transcripts in wild type and *rlsb*-silenced plants, it is apparent that silencing led to greatly and correspondingly reduced levels of accumulation for both transcripts (Figure 8D). In the silenced plants, both mRNAs showed correlating ratios of reduction, with approximately 25–50 fold lower levels (*rlsb*-silenced 1 and 2, respectively), relative to wild type for both *rbc*L and *RLSB*. The fact that both of these transcripts were detectable, even at greatly reduced levels, indicates that *RLSB* expression was not completely silenced. The finding that *rbc*L mRNA and LSU protein levels were reduced in close coordination with levels of silencing for *RLSB* provides support for our hypothesis that this S1-RNA binding protein affects *rbc*L gene expression, at least in part, at the levels of translation and mRNA accumulation.

Of four other representative plastid-encoded proteins examined in the *rlsb*-silenced *Arabidopsis*, *chloroplast coupling factor 1* (CF1αβ alpha and beta subunits) showed an intermediate silencing-associated reduction in accumulation (less than that of LSU, approximately 10 – 12 fold), while PsaC and PsbA levels were mostly unaffected (Figure 8C). As expected, immunoblots confirmed that the nuclear-encoded, plastid targeted SSU was also greatly reduced in the silenced lines; this methodology did not detect any SSU protein in the silenced plants. Another nuclear-encoded, plastid-targeted protein, the Cp28 RNA binding protein [16,40], was not affected by silencing of *RLSB*. Two nuclear-encoded cytoplasmic proteins, PEPCase and NAD-dependent malic enzyme (NAD-ME), showed no reductions in the silenced plants (Figure 8C). In fact, NAD-ME showed a slight increase in abundance (approximately 3-fold) relative to control plants.

Taken together, the data shown in Figure 8 indicate that *RLSB* gene expression was greatly reduced, but not completely eliminated, due to incomplete gene silencing in these *Arabidopsis* lines. This resulted in a corresponding reduction in levels of *rbc*L mRNA and LSU protein, providing strong evidence that the nuclear-encoded RLSB protein is necessary for normal levels of *rbc*L gene expression in the chloroplasts of this C_3_ plant.

Confirming evidence that RLSB affects *rbc*L expression was obtained from studies that were initiated using *Arabidopsis* lines containing a T-DNA insertion within the At1g71720 locus that encodes the RLSB protein in this plant. The data shown in Additional file 8: Figure S8 summarizes findings from one of these lines (SALK_015722, identified through The *Arabidopsis* Information Resource TAIR and obtained through *Arabidopsis* Biological Resource Center (ABRC; http://abrc.osu.edu) [41]. The T-DNA insert in this line is within the region encoding the S1 RNA binding domain, which would be expected to eliminate the binding ability and function of this protein. Plants homozygous for the T-DNA insert in this line were never recovered. All of the heterozygote siblings from line SALK_015722 showed reduced accumulation of *rbc*L-encoded Rubisco LSU protein (Additional file 8: Figure S8A), when compared to Col0 plants, or sibling plants that segregated out the insert (Additional file 8: Figure S8B). Note that in these At1g71720 insertion mutants, levels of LSU were not reduced as dramatically as in the *rlsb*-silenced plants (approximately 5-fold, as opposed to 25–50 fold in the *rlsb*-silenced plants). Also unlike the *rlsb*-silenced *Arabidopsis*, levels of *rbc*L mRNA were not reduced in any of these lines (Additional file 8: Figure S8C), suggesting that in this case, translation of *rbc*L mRNA, but not mRNA stability, was impeded. It should be noted that these lines did not stably maintain the T-DNA insert, so plants heterozygous for the insert in the At1g71720 locus were recovered only rarely, and then not at all after four self-pollinated generations. For this reason, these T-DNA insertion lines were not extensively analyzed, and our focus shifted to the *rlsb*-silenced lines for the more detailed analyses of RLSB function in *Arabidopsis* as presented in Figure 8.

## Discussion

### The RLSB binding protein in maize and other plants

Findings presented here indicate that RLSB is a nuclear-encoded S1-domain RNA binding protein that interacts with plastid-encoded *rbc*L mRNA, thereby activating or enhancing *rbc*L gene expression. RLSB was purified from chloroplasts based on its specific binding to the 5’ region of *rbc*L mRNA *in vitro*. The purified protein is highly conserved among a wide variety of monocot and dicot C_3_ and C_4_ plant species, and contains a predicted plastid transit sequence. It is localized to chloroplasts in both the C_3_ dicot *Arabidopsis* and the C_4_ monocot maize. Most significantly, in the leaves of all three C_4_ species examined, it co-localized with Rubisco only within BS cell chloroplasts, corresponding with the specific cellular compartmentalization of Rubisco in C_4_ leaves.

RLSB is an S1 binding domain protein in the same category as the ribosomal protein S1, from which this class is named [44]. Other than its conserved binding domain, RLSB is a unique chloroplast protein; it shows very little overall identity with known examples of plastidic ribosomal S1 proteins, including those from spinach (AAA34045.1), cucumber (ABK55725.1), rice (ABF95618.1) or *Chlamydomonas* (CAE51165.1). Stretches of amino acids spanning the S1 RNA binding domain display 33% - 73% maximum identity with gaps, depending on the species comparisons. However, outside of this conserved domain there are no extensive regions of significant similarity between the plastidic RLSB and ribosomal S1 proteins. RLSB orthologs in *Arabidopsis*, maize, and other plant species used for our comparisons (Additional file 1: Figure S1, Additional file 2: Figure S2) appear to be unique members of the S1 class of RNA binding proteins, distinct from other known proteins of this class, and from other known plastidic RNA binding proteins.

The S1 binding domain that distinguishes this protein is found in a large number of RNA binding proteins [44]. The S1 binding domain structure is very similar to that of cold shock proteins, and appears to be derived from a very ancient class of nucleic acid binding proteins. While these proteins are known to be widespread among a variety of organisms, there is very little known about the function of proteins that contain this domain. In higher plants, some non-ribosomal proteins known to possess S1 domains include the plastidic polynucleotide phosphorylase [45], RNase E/G-type endoribonuclease [46], and exosome subunit AtRrp4p [47]. Examples of other known S1-domain proteins include transcription factor NusA and polynucleotide phosphorylase in bacteria [48,49], and a nucleic acid binding protein of unknown function in humans [50].

Analysis by immunolocalization as well as mechanical cell-separation demonstrated the BS-specific localization of RLSB in leaves of the C_4_ plant maize. Maize proteomic data localizes RLSB to the chloroplast nucleoid, where transcription is coupled to post-transcriptional RNA processing and translation [51]; http://ppdb.tc.cornell.edu/dbsearch/gene.aspx?id=674610. This is a very comprehensive database, however it does not provide information about the occurrence of RLSB in separated BS or M cells. This information is provided for other C_4_ proteins (for example, see Rubisco LSU, http://ppdb.tc.cornell.edu/dbsearch/gene.aspx?id=652357). Protocols for separating BS and M cells have been shown to affect the accumulation of some BS and M proteins in C_4_ plants (for example, [52]), and it is possible that RLSB was not present when the separated BS and M extracts were used for proteomic analysis. The absence of RLSB from the separated cell populations might be related to our observation that this protein does not appear to be stable in the disrupted BS cells after separation by leaf rolling. This is why the BS-enriched strands were used immediately for protein isolation, without further purification of the BS strands after the M cell “roll out” (Figure 2).

This current study is focused on RLSB protein, and not its mRNA. Still, it is important to mention that our experimental findings of BS specificity for RLSB protein in maize might not appear to be in agreement with data contained in two recent maize transcriptome databases (30, 33), which have analyzed mRNA populations in separated BS and M cells. For example, using the B73 C3/C4 transcriptome web browser tool (http://c3c4.tc.cornell.edu/search.aspx) of Li et al. [30] for *RLSB* (GRMZM2G087628) mRNA, only a portion of the transcript sequence is indicated as being present in both the laser capture microdissection (LCM) leaf tip (mesophyll) and LCM leaf tip (bundle sheath) graphs from that database. The first four exon sequences from the 5′ portion (more than half of the full-length mRNA sequence) are missing from these two graphs; only 3′ exon sequences are indicated as being present in the two separated cell types. This might imply an anomaly for *RLSB* mRNA in the separated cell populations. In contrast, transcriptome graphs from leaf tip and leaf base (cells not LCM separated, combined transcripts from both cell types) show all eight exon sequences present. In stronger contradiction to our RLSB protein data, the transcriptome database of Chang et al. [33] (based on enzymatic digestion-mechanical separation instead of LCM) actually indicates that GRMZM2G087628 transcript sequences are significantly more abundant in M cells than in BS cells (>13 fold). These databases are both very comprehensive and useful tools for analysis of C_4_ gene expression. However, for reasons stated above, it is possible that the integrity/cell specificity for this particular mRNA was not maintained during the cell separation protocols utilized for those databases, leading to conflicting findings. Alternatively, post-transcriptional control of *RLSB* mRNA processing, stability, or translation could be involved in determining cell-type specificity for the RLSB protein, as has been found for genes encoding Rubisco and many other C_4_ proteins [5-7,11]. An analysis of *RLSB* mRNA transcription, accumulation, and stability in BS and M cells is currently under investigation and will be included in a separate study.

In *Arabidopsis*, the eFP browser (http://bbc.botany.utoronto.ca/efp/cgi-bin/efpWeb.cgi) shows a strong correlation in the timing of expression of mRNA accumulation, primarily in photosynthetic (green) plant tissues, for *RLSB* (At1g71720) (http://www.bar.utoronto.ca/efp/cgi-bin/efpWeb.cgi?dataSource=Developmental_Map&modeInput=Absolute&primaryGene=At1g71720%20&secondaryGene=None&modeMask_low=None&modeMask_stddev=None) and *rbc*L (Atcg00490) (http://www.bar.utoronto.ca/efp/cgi-bin/efpWeb.cgi?dataSource=Developmental_Map&modeInput=Absolute&primaryGene=Atcg00490%20&secondaryGene=None&modeMask_low=None&modeMask_stddev=None), providing additional correlative evidence for RLSB and *rbc*L interaction. The online expression profiles indicate that in *Arabidopsis,* an exception to the *RLSB* and *rbc*L correlation occurs in dry seed, where transcripts for *RLSB*, but not *rbc*L, occur in abundant amounts. Rubisco mRNAs and enzymatic activity have been found to occur transiently during very early seed development, dropping off at later stages [53,54]. During seed development in *Brassica napus*, Rubisco activity, independent of the calvin cycle, has been shown to enhance carbon acquisition, which promotes the formation of seed oil [55]. In addition, mRNA accumulation and translation of the LSU and SSU proteins can occur very soon after germination [5,8,56-58]. Thus, there is a potential role for RLSB during seed development, and possibly for enabling the rapid onset of early Rubisco synthesis during germination.

### RLSB binding activity, and *rbc*L gene expression in maize and *Arabidopsis*

RLSB was isolated based on its ability to bind *rbc*L 5′ RNA but not a control RNA *in vitro*, indicating at least some specificity for the Rubisco chloroplast transcript. Further analysis by RIP/qRT-PCR extended these initial findings by demonstrating prominent selective binding of RLSB to *rbc*L mRNA, but not other plastid-encoded transcripts, *in vivo*. The *in vivo* assay also revealed the possibility of much weaker interactions with *psb*A, *atp*A, and *atp*B mRNAs, all of which accumulate in both BS and M plastids of maize leaves, with *psb*A actually being far more abundant in M plastids [39,59]. The fact that RLSB was not detectable in maize M cells would suggest that this greatly reduced binding activity to mRNAs other than *rbc*L might not be biologically significant, likely caused by background binding within the chloroplast lysates. The data presented here cannot rule out the possibility that RLSB may in fact have additional target RNAs within BS chloroplasts that were not identified because they were not included in this assay. Higher plant chloroplast genomes can encode over two hundred protein-encoding and non-coding mRNAs [6,60,61]. A genomics-based search for additional mRNA targets in maize and *Arabidopsis* plastids is required to definitively identify the full range of RLSB binding specificity, and is currently in progress. However, the finding that RLSB interacted preferentially with *rbc*L mRNA, and only weakly or not at all with any of the other six plastid-encoded mRNAs examined, indicates a very high degree of binding selectivity.

In maize, the developmentally early lower regions of *rlsb-1*/*rlsb-2* leaves displayed greatly lowered RLSB protein accumulation, together with reductions in levels of *rbc*L mRNA, as well as in the accumulation and synthesis of the LSU protein. All of these mutation-associated changes (*rbc*L mRNA, LSU accumulation and synthesis) correlated very closely in these same lower leaf regions. These parameters all recovered to normal non-mutant levels in the developmentally advanced outer regions. Thus, in maize *rlsb-1/rlsb-2* mutants, RLSB production, and associated LSU synthesis/accumulation, were delayed (but not eliminated) along the maize leaf developmental gradient. In comparison, *rlsb*-silencing in the C_3_*Arabidopsis* plant led to greatly reduced levels of *RLSB* mRNA and its encoded protein throughout the entire length of the leaf, relative to wild type Col0. Throughout the same silenced leaves, strongly correlating reductions in *rbc*L mRNA and LSU protein also occurred.

When comparing the effects of reduced RLSB expression in the silenced *Arabidopsis* and transposon-mutagenized maize, some similarities and significant differences become apparent. In both the C_3_ and the C_4_ experimental plant systems, reductions in levels of *RLSB* and *rbc*L mRNA, as well as their encoded proteins, occurred in approximate coordination (Figures 5 and 8). Such findings support a common regulatory connection between RLSB and levels of *rbc*L gene expression in both plants. As in *RLSB/RLSB* maize, Col0 *Arabidopsis* leaves had more abundant levels of *rbc*L mRNA than *RSLB* mRNA (Figures 5B and 8D, note the difference Y-axis scales). However, in *Arabidopsis* this difference was less pronounced (25–50 fold, relative to more than 150-fold in maize). In maize, insertion mutagenesis did not completely eliminate *RLSB* expression, allowing for reduced but detectable levels of *rbc*L expression in lower *rlsb-1*/*rlsb-2* leaf regions. Silencing of *RLSB* in *Arabidopsis* resulted in much more dramatic effects than in the maize mutants, but very low levels of *RLSB* and *rbc*L expression were still detectable in these plants.

Lowered *RLSB* expression in both plants led to corresponding coordinated effects on levels of *rbcL* mRNA accumulation, LSU synthesis (in maize), and LSU accumulation. Lowered levels of *rbc*L mRNA in *RLSB*-silenced *Arabidopsis* and *rlsb-1/rlsb-2* maize mutants suggest that RLSB is required for the stabilization of these transcripts. However, the finding that LSU synthesis and accumulation were reduced much more dramatically than *rbc*L mRNA in *rlsb-1/rlsb-2* lower leaf regions (approximately 4-fold for mRNA, 15 fold for protein), and reduced LSU accumulation was not accompanied by lower *rbc*L mRNA in the *Arabidopsis* At1g71720-insertion heterozygotes, suggests a role in translation as well. Similar to the data of Figure 5B, an earlier study also found that *rbc*L mRNA accumulation in maize was reduced approximately four-fold in response to a decrease in translation [38]. In fact, many studies have confirmed a close relationship between the processes of transcript stabilization and translation in chloroplasts, with both processes regulated by RNA binding proteins [5,16,17,24,62]. Taken together with the *in vitro* and *in vivo* binding data, evidence presented here clearly implicate RLSB as a key determinant of *rbc*L gene expression in the chloroplasts of all photosynthetic leaf types in C_3_*Arabidopsis*, and exclusively in the BS chloroplasts of C_4_ maize. The tight correlative changes associated with lowered *RLSB* expression (confirmed by multiple levels of analysis) are strongly indicative of a regulatory link between RLSB and *rbc*L gene expression, with RNA binding possibly implementing an effect at the level of RNA metabolism (*rbc*L mRNA translation and stability).

A significant difference between the two plant systems was apparent when comparing the effects of reduced RLSB on proteins other than LSU (Figures 6 and 8E). Although there were no effects on nuclear-encoded PEPCase or CP28 in either plant, effects on other representative plastid- and nuclear-encoded proteins differed considerably. In contrast to maize, reduced RLSB in *Arabidopsis* was not associated with any changes in the accumulation of the plastid-encoded PsaC or PsbA (components of PSI and PSII, respectively), while reductions in CF1αβ were considerably less pronounced. The nuclear-encoded NAD-ME actually increased in the *rlbs*-silenced *Arabidopsis*, as opposed to the strong decrease observed in the maize mutants. It might be expected that any direct effects of reducing this highly conserved S1 binding protein would be consistent between the C_3_ and C_4_ plants, with shared reductions in LSU being distinctly prominent. However, with regards to effects on other proteins, variations between two photosynthetic systems proteins were clearly evident.

### Other effects of reduced *RLSB* expression

In maize, reduced and delayed RLSB and LSU production in *rlsb-1/rlsb-2* lower leaf regions was associated with strong decreases in the accumulation of several additional plastid- and nuclear-encoded proteins. Reductions in levels of the nuclear-encoded SSU protein were expected, since the expression, synthesis, and assembly of the two Rubisco subunits are tightly coordinated [7,20,63]. It was, however, surprising to observe such strong reductions in other representative plastid- and nuclear-encoded proteins. Like LSU, these all increased to normal levels in outer regions of the leaf. Developmental increases in *rbc*L and *RLSB* expression were also associated with basipetal recovery of the virescent phenotype in *rlsb-1*/*rlsb-2* leaves. Multiple effects similar to those in the *rlsb-*1/*rlsb-*2 mutants have been associated with other maize mutations that affect general regulators of plastidic gene expression, such as *cps* and *hcf*[38]. However, there are several characteristics and observations that distinguish the *rlsb* mutants from the general regulator mutants. First and most importantly, findings presented here clearly demonstrate that RLSB is strictly confined to chloroplasts within the maize leaf BS cells. However, most of the affected plastid-encoded proteins actually accumulate equally, or even more abundantly, within M cell chloroplasts of wild type maize leaves [39,59]. A general regulator that is specifically localized within BS chloroplasts could not directly affect translation of mRNAs in M chloroplasts. Second, while severe reductions in PsaC and PsbA were associated with decreased *RLSB* expression and LSU production in the lower leaf regions of *rlsb*-1/*rlsb*-2 maize mutants, these same proteins were not affected at all in the *rlsb*-silenced *Arabidopsis*, even in the presence of much more severe RLSB and LSU decreases. A highly conserved general regulator of ribosome assembly would be expected to have the same function and produce analogous effects in both plants. Third, there was no significant decrease in plastid ribosomal RNA levels observed for the *rlsb-1/rlsb-2* mutants, indicating that, unlike the *cps* and *hcf* mutants, ribosome accumulation was not affected. Fourth, in contrast to the *cps* and *hcf* mutants that affect only translation, the *rlsb-1/rlsb-2* mutants showed variation in accumulation levels for several plastid mRNAs as well. Fifth, unlike the general plastidic regulators, the effects of *rlsb-1/rlsb-2* mutants were not confined to the chloroplasts; levels of two nuclear-encoded proteins were also significantly reduced, including the NADP-ME that was not affected in *cps* or *hcf* mutants [38]. It is clear that RLSB shows selective binding to *rbc*L mRNA in affinity purification and in RIP/qRT-PCR analysis; binding to other plastid-encoded transcripts, including those with severely reduced protein accumulation in lower *rlsb-1*/*rlsb-2* leaf regions, was minimal or did not occur. These distinguishing characteristics, together with the BS-specific localization of RLSB, its selective binding to *rbc*L mRNA and clear effects on LSU production, confirm the distinct identity of this protein, functionally separating it from the previously identified plastid ribosome assembly mutants of maize.

All of the recovered *rlsb-*silenced *Arabidopsis* had multiple developmental abnormalities, including dark purple leaves, reduced leaf size, and impaired root development. They did not produce bolts or flowers, and died after 30 days. These effects were not observed on non-transformed plants grown without selection on the same medium under the same conditions (Additional file 4: Figure S4, bottom panels). These effects were also not observed in any of the heterozygous At1g71720-insertion plants, which showed much less severe reductions in LSU accumulation. While anthocyanin production is known to increase when *Arabidopsis* plants are grown in the presence of high sucrose [64,65], leaves of the *rlsb*-silenced plants had much darker pigmentation than leaves of non-transformed plants grown on the same high-sucrose media. The very high levels of purple pigmentation is an indicator of a stress response [64]. It is clear that in this C_3_ plant species, severely lowered levels of RLSB not only leads to greatly reduced production and accumulation of Rubisco, but also affects many other aspects of growth and development.

The more severe reduction in *RSLB* expression in *rlsb*-silenced *Arabidopsis* correlated with a much greater effect on LSU mRNA and protein accumulation than in *rlsb*-1/*rlsb*-2 maize leaves. Based on the sampling of proteins shown in Figure 8, it appears that the greatly reduced *RLSB* expression did not result in a strong general cessation/decrease of proteins other than LSU and its associated SSU in this C_3_ plant. Like increased anthocyanin, increases in the nuclear-encoded mitochondrial enzyme NAD-ME are often occur in response to plant stress [5,66,67], in this case possibly brought about by growth/developmental challenges in the silenced *Arabidopsis*.

Several studies have also demonstrated that plants with reduced Rubisco are recoverable and viable [63,68-72]. In some cases these plants can have their growth enhanced by supplementing atmospheric CO_2_[69], or for the *rlsb-*silenced *Arabidopsis* described here, by adding additional carbon source to the media to facilitate more heterotrophic growth. In contrast to the multiple associated effects reported here for the *rlsb*-1/*rlsb*-2 mutants, a recent study with a different maize mutant showed that greatly reduced Rubisco accumulation, caused by a defect in the assembly factor RAF1, did not show any effects on other plastid-encoded proteins [72]. It may be important to consider that separate analysis of lower and upper leaf regions were not reported in that study, and the developmental stages analyzed might not be directly comparable to those used here. However, the finding that greatly reduced Rubisco within the *raf*1 mutant leaves had no observable effect on any other plastid-encoded proteins represents a clear difference from the effects observed in lower regions of the *rlsb*-1/*rlsb*-2 leaves. It could be relevant that RLSB impacts the earliest step of LSU synthesis, whereas RAF1 affects Rubisco accumulation at the much later step of Rubisco assembly/degradation.

At this stage, we can only speculate about mechanisms by which reduced *RLSB* expression in maize would have such a dramatic effect on the production of several plastid- and nuclear-encoded proteins other than LSU and SSU, especially with regards to genes not encoded within BS chloroplasts. A broad-range coordinated impact on multiple photosynthetic genes is certainly not unique to the *rlsb*-1/*rlsb*-2 effects presented here. Numerous studies have demonstrated that photosynthetic metabolism affects plastid gene expression at the levels of transcription, translation, as well as assembly/stabilization of PSI, PSII and PET complexes [6,17,73,74]; many photosynthesis-associated nuclear genes are affected as well [6,73-76]. A few notable examples of widespread regulation of photosynthetic genes, mediated through transcriptional or post-transcriptional regulation, occur during high light stress [77], photomorphogeneis [78], and stress-induced mRNA decay [79]. Photosynthetic signaling-regulatory networks occur within and outside of chloroplasts, involving close interactions between cells and different cellular compartments. These networks extend to mitochondria, where NAD-ME is localized [75,80-82]. Lower levels of Rubisco and the resulting impact on carbon fixation would redirect photosynthetic electron transfer, leading to the generation of redox signals such as reactive oxygen species, hydrogen peroxide (H_2_O_2_) and O_2_ itself [73,74,77,83,84]. Other processes known to regulate photosynthetic genes, such as the rate of CO_2_ fixation, carbon metabolism, photosynthetic intermediates, and sucrose accumulation [5,6,80,82,85,86] could all be impacted by reduced Rubisco production in *rlsb-*1/*rlsb-*2 maize mutants and *rlsb*-silenced *Arabidopsis.* In consideration of these effectors, it is perhaps not too surprising that inhibition of LSU synthesis early in the development of the C_4_ maize leaf might signal suppressive effects on the expression of other genes related to photosynthetic function or metabolism, even in other cell types and cellular compartments. Affected genes in the *rlsb*-1/*rlsb*-2 mutants include redox and sink-responsive light-reaction genes in BS and M cells, the nuclear-encoded plastid-targeted decarboxylating NADP-ME in BS cells, and the mitochondrial NAD-ME. While little is known about the regulation of metabolic (non-photosynthetic) NAD-ME in NADP-ME-type C_4_ plants such as maize [81], it is clear that this protein, which is encoded, translated, and functions outside of the chloroplasts, was also greatly reduced in the *rlsb-1/rlsb-2* mutants. Also unclear is why associated effects on some proteins were severe in the C_4_ leaves of maize, but for the most part did not occur in the C_3_ leaves of *Arabidopsis*. Differences in the severity of RLSB-associated effects in maize and *Arabidopsis* could be related to the more complex, multi-compartmental regulatory interactions required for energy-intensive C_4_ differentiation, abundant gene expression/protein accumulation, and enhanced photosynthetic capacity [5,11,82].

For all of the experiments described above, we never recovered any *rlsb*-silenced lines of *Arabidopsis*, homozygous *Arabidopsis* At1g71720 T-DNA insertion mutants, or any *rlsb-1/rlsb-2* maize lines, in which RLSB or LSU had been completely eliminated. Incomplete silencing, heterozygosity, and partial suppression of mutator activity all appear to have allowed for low levels of *RLSB* expression in each of the experimental systems used here. We expect that complete absence of RLSB would result in a complete absence of LSU production, severely reducing seed or seedling viability.

The progressive base to tip variation in phenotype (virescent to green phenotype, increases in synthesis and accumulation of LSU and other proteins) observed in *rlsb-1*/*rlsb*-2 leaves is not unique. A recovery to wild type phenotype also occurred at the leaf tip of the transposon-based *bundle sheath defective* (*bsd*1, an ortholog of *G2*) maize mutants [87]. It is likely that the much lower levels RLSB and LSU produced within *rlsb*-1/*rlsb*-2 lower leaf regions, relative to the same region of *RLSB/RLSB* leaves, caused a delay but not a cessation of full photosynthetic development for these leaves. Significantly lowered production would be expected to cause a slower buildup of both proteins in the less advanced lower region, relative to the much more rapid accumulation that would normally occur in the same region of wild type leaves. In this scenario, reduced RLSB and LSU production would slow their rates of accumulation along the length of the maize leaf developmental gradient. Eventually, levels of accumulation would “catch up” to normal *RLSB*/*RLSB* levels, but much further up along the developmental gradient. The more gradual increase along the length of the gradient would eventually allow both protein to reach wild type levels, but only in the older developmentally advanced upper regions of *rlsb*-1/*rlsb*-2 maize leaves.

It is known that transposon insertion within a gene does not always lead to the complete elimination of the gene’s activity [88]. In the case of Mu, there are several potential causes for partial suppression of mutator activity. Although somatic excision could lead to recovery of expression [89], there was no detectable excision from the *RLSB* gene at the leaf base, or more significantly, at the leaf tip (not shown). There are also potential epigenetic effects from insertions near promoters or introns [88-90]. Both the *rlsb-1* and *rlsb-2* insertions were within the first exon, 8 and 45 nucleotides, respectively, of the first 5’ splice junction. This localization presents a likely mechanism, which is the use of an in-frame 5’ donor site within the Mu transposon. Use of the Mu donor site can cause alternative splicing [88,89,91]. In this case, removal of transposon sequence from some *rlsb-1* or *rlsb-2* transcripts might have caused minor changes (such as a small deletion) within mRNA sequences encoding the RLSB N-terminal region, leading to reduce production, accumulation, and/or functionality. Additional studies will be required to determine exactly how these Mu insertions affect RLSB function in these mutant plants.

To understand the elusive molecular processes that mediate BS or MP cell-specific gene expression in C_4_ plants, and how such processes might have developed from pre-existing C_3_ forms, it is essential that gene-specific trans-acting regulatory factors with properties unique to this photosynthetic pathway be identified [5,7,11,12]. In C_4_ leaves, such factors would be expected to show localization or activity that is specific to only one of the two specialized photosynthetic leaf cell-types. It is likely any trans-acting factors responsible for the highly specific C_4_ expression patterns would not have originated *de-novo*, but would actually occur and be functionally present in a “default” expression mode in C_3_ plants [5,7,11]. The modification of RLSB binding activity to BS cell-specificity in C_4_ plants would not require any changes to the *rbc*L gene itself. In fact, regulatory regions of these plastidic genes are highly conserved across C_3_ and C_4_ plant species [92]. The strict correlation between RLSB and *rbc*L gene expression, occurring at several levels as determined by multiple levels of analysis, suggests that BS-specific accumulation of the RLSB binding protein to *rbc*L mRNA may be one determinant leading to the BS-specific localization of Rubisco that is characteristic of kranz-type C_4_ plants.

The mechanism(s) by which RLSB post-transcriptionally activates or enhances *rbc*L gene expression in C_3_ and C_4_ plants is under investigation. Plastidic mRNA binding proteins often function in association with other proteins; in fact, the *rbc*L mRNA-based affinity purification of several proteins along with RLSB provides evidence that this protein may function as part of a larger protein complex. How this and any associated proteins had their expression modified to become BS specific during the evolution of C_4_ capability will involve “going back a step” from our traditional levels of analysis, focusing on the cell-type specific regulatory gene (*RLSB*), instead of the cell-type specific gene (*rbc*L) at the end of a proposed and possibly extensive regulatory chain. Previous studies of C_4_ gene expression have revealed a complex regulatory system, involving multiple levels of transcriptional, post-transcriptional, and post-translational control, all integrated together to achieve full C_4_ photosynthetic capacity. There is no evidence for a “global regulator” of C_4_ expression; genes encoding different photosynthetic enzymes show unique patterns of expression indicative of independent regulation [5]. RLSB, as a regulator of *rbc*L gene expression, provides a new insight into how one component of this extensive photosynthetic regulatory system might have been modified from an original C_3_ “default” function to perform the same function, but in a more specialized fashion, in C_4_ plants.

## Conclusions

RLSB was isolated from chloroplasts of a C_4_ plant by affinity-purification based on its ability to bind *rbc*L mRNA *in vitro*. This protein, encoded by the nuclear *RLSB* gene, contains an S1 nucleic acid binding domain and is highly conserved among a wide variety of C_4_ and C_3_ plant species. RLSB contains a plastid transit sequence, and co-localizes with LSU to chloroplasts. Most significantly, it accumulates specifically within the BS chloroplasts of maize and other C_4_ plants. In maize chloroplasts, RLSB showed selective binding to *rbc*L mRNA, but not to other representative plastid-encoded transcripts. In maize *RLSB* insertion mutants, RLSB accumulation was reduced along the leaf developmental gradient, with lowest levels at the leaf base, and increasing levels toward the leaf apex. Delayed/reduced RLSB accumulation led to corresponding reductions in *rbc*L mRNA accumulation as well as synthesis/accumulation of LSU protein, indicating regulatory functions at the levels of mRNA stability and translation. Other developmental effects included virescent/yellow leaves and reductions in several additional proteins (including some PSI and PSII components) that locate to other cellular compartments and cell types of C_4_ leaves. Effects such as these were likely associated with decreased photosynthetic function and disruption of associated signaling networks. Reduction of RLSB production in *Arabidopsis* by RNA silencing or insertion mutation revealed that, as in C_4_ maize, RLSB affects *rbc*L gene expression at the levels of mRNA and protein accumulation in the chloroplasts of this C_3_ plant. While reduced RLSB in both maize and *Arabidopsis* lead to corresponding decreases in *rbc*L mRNA and LSU protein, secondary effects (reductions for other proteins) in *Arabidopsis* were not as severe as in maize. The strict co-localization to Rubisco containing chloroplast and tight correlation with *rbc*L gene expression suggests RLSB may play an essential role in determining photosynthetic gene expression in all plants, and possibly contribute to BS-specific localization of Rubisco in C_4_ plants.

## Methods

### Plant material and growth conditions

*Amaranthus hypochondriacus* var RI03, *Flaveria bidentis*, *Setaria viridis*, and *Arabidopsis* plants were grown in artificial soil in a greenhouse, or in a growth chamber in soil or on media (see below) using growth conditions described previously [56,67,93]. Maize (*Zea mays*) lines containing the *rlsb*-insertion (*rlsb-1*/*rlsb-2*, *rlsb-1*/+ and *rlsb-2*/+), and sibling lines lacking the *rlsb Mu* insert (*RLSB/RLSB*), as well as wild type maize line B73 [32] were grown in artificial soil in a growth chamber (14 h/d illumination at 170–200 mmol photons m^-2^ s^-1^, with 22°C day 19°C night temps).

### RNA affinity purification of RLSB protein from chloroplasts extracts

Extracts for RNA binding were prepared from purified amaranth chloroplasts as described [26]. Extracts used for purification were approximately 1 – 2 mg protein per ml, as determined using a Bradford protein assay (Bio-Rad).

*In vitro* transcriptions and acrylamide gel purification of RNAs corresponding to the 5′ region of the amaranth *rbc*L mRNA (beginning at the −66 processed end of mature *rbc*L transcript, and ending at the +60 position) and control 7z-AS RNA (a 130 nt 3’ UTR from a yeast viral RNA [27]), were performed using DNA templates and methods described previously [26]. The *rbc*L 5’ and control RNA was labeled with biotin at its 3’ end using Biotin hydrozide (EZ link Biotin-LC-hydrozide, Thermo Scientific) according to manufactures protocols (http://piercenet.com/instructions/2160124.pdf). The 3′-biotinylated RNA was ethanol precipitated twice, resuspended in RNAse-free H_2_O, and stored at −80°C.

For each RNA affinity purification, 50 μl of streptavidin magnetic beads (New England Biolabs) were used. Beads were separated from supernatants using a magnetic stand (New England Biolabs). The beads were first washed twice with binding buffer (40 mM KCl, 10 mM MgCl_2_, 3 mM dithiothreitol, 0.05 mM EDTA, 8.5% (v/v) glycerol, 10 mM HEPES, pH 7.9, and at least 0.5 mg/μl of tRNA as a nonspecific competitor), and then incubated with either 2 ml biotinylated 5′*rbc*L or biotinylated 7z-AS RNA (approximatey 25 picomoles per ml) for 10 minutes. The RNA coated beads were then washed twice with binding buffer.

Extracts for binding reactions were adjusted to 1X binding buffer and 0.5 μg/μl tRNA, and pretreated with buffer-washed beads (200 μl of plastid extract to 50 μl beads) prior to use in binding reactions (beads used for this pretreatment were discarded). Pre-treated extract (80 μl) was then added to each 50 μl aliquot of RNA coated beads, and the binding reactions were incubated at room temperature for 15 minutes. The beads were then washed twice with binding buffer. Washed beads were treated with 2 μl of RNAse cocktail (Ambion) for 15 minutes at room temperature, and loaded onto a 22 cm long 10% SDS-PAGE gel. Bands of interest visualized by silver staining were cut from the gel and subjected to trypsin digestion and Maldi-Tof mass spectrometry (Custom Biologics, Toronto, CA).

### Antibodies

Antisera against conserved peptide sequences within the *Arabidopsis* RLSB protein were produced in rabbits using the peptide sequences shown in Figure 1C (Proteintech Group, Inc., Chicago, IL, http://www.ptglab.com). This antisera was affinity purified using the 149 aa peptide that is shown in Figure 1C, using an UltraLink iodoacetyl micropeptide coupling kit (Thermo Scientific). The 149 aa peptide itself, containing a T7 tag, was expressed in *E. coli* using the pET-17b expression vector, and purified using a T7∙Tag Affinity Purification kit (Novagen).

Antibodies against the Rubisco large subunit (LSU), PEPCase, and NAD-ME have been described [66,94,95]. Antisera against the plastid proteins PsaB, PsaC, PsbA, and AtpB were obtained from Agrisera (Vännäs, Sweden, http://www.agrisera.com). Antisera against Cp28, CF1αβ (this antisera reacts to both the alpha and beta subunits), and NADP-ME were the generous gifts of Masahiro Sugiura (Nagoya University, Nagoya Japan), Julian Hibbard (University of Cambridge, UK), and Richard Leegood (University of Sheffield, UK), respectively.

### Immunolocalization

Immunolocalizations were performed using paraffin-embedded leaf sections, as described [94,95]. *Arabidopsis* leaf sections were taken from fully expanded leaves of Col0 plants, midway between the apex and base. Leaf sections of maize line B73 were taken from first or second 10 – 14 cm long leaves, approximately midway between the apex and the base. Sections from *Flaveria bidentis*, *Setaria viridis*, and *Arabidopsis* plants were taken from young leaves between one third to one half full expansion, approximately midway between the apex and base. Sections were reacted with the primary antisera indicated in the Figures, and then secondary antisera conjugated with R-phycoerythrin or Alexafluor 546 (Molecular Probes/Life Technologies, Carlsbad, California). Epifluorescence images were captured using a DM IRE2 inverted compound microscope (Leica Microsystems, Wetzlar, Germany) equipped with fluorescent and brightfield imaging systems, and a Retiga Exi cooled CCD camera (Q Imaging, Burnaby, BC, CA). Confocal images were captured using an LSM710-InTune Confocal (inverted) Microscope System with Zen Imaging software (Carl Zeiss MicroImaging), using a 20X or 40X objective; Alexafluor 546 excitation was done using a 529 nm laser line, and fluorescence was collected at 564–577 nm.

### Silencing of *RLSB* in *Arabidopsis*

Primers corresponding to a 447-nt region of *the Arabidopsis RLSB ortholog* (Figure 1, Additional file 9*, accession #*JX843767*)* (a region unique to RLSB to eliminate off-target silencing) was inserted as an inverted repeat into the pHannibal silencing vector, and then mobilized into the pART27 binary vector [96]. Six-week-old *Arabidopsis* Col-0 plants were transformed using floral dip [97]. Agrobacterium treated seeds were surface-sterilized using vapor-phase sterilization, and germinated on solid MS medium with 3% sucrose. Germination and the first 14 d of growth occurred under 16-h illumination at 22°C in Petri dishes containing kanamycin (100 mg/L), after which resistant seedlings were transferred to and sterilely maintained in PlantCon tissue culture containers (MP Biomedicals) on MS medium with 8% sucrose, without selection. Potential *rlsb-*silenced lines were harvested and assessed (using PCR to check for correct inserts and qRT-PCR to confirm reduced *RLSB* mRNA levels) at 6–8 weeks. Six of the confirmed *rlsb*-silenced plants were selected for further analysis; data from two of these plants (designated *rlsb-*silenced 1 and 2) are shown in Figure 8, panel D. As controls, non-transformed seed from wild type Col0 plants were germinated and grown, and transferred using the same media and growth conditions, except without initial Kanamycin selection (Additional file 4: Figure S4, bottom panels).

### Mutant maize lines

Mu insertions in the maize *RLSB* gene *(rlbs1 and rlbs2)* were identified in a maize line with active *Mutator* (*Mu*) transposons by screening with oligonucleotides based on the maize ortholog of *RLSB* (accession #JX650053) and both borders of the Mu insert (Additional file 3: Figure S3 and Additional file 9). The mutation is inherited as a single, recessive Mendelian trait. It’s effects were visualized in seedlings with virescent 1^st^, 2^nd^, and 3^rd^ leaves, which gradually green starting at the tip and progressing in the basepetal direction (Additional file 4: Figure S4, top panels). No virescence was observed in wild type plants grown under the same conditions. Unless noted, leaf material from the mutant plants was isolated from 6 – 8 cm 2^nd^ or 3^rd^ leaves.

### Protein accumulation and synthesis

Soluble or membrane-bound protein extracts were prepared as described [57,67,95]. Total maize leaf protein extracts were prepared according to the methods of [98]. A mechanically separated and purified population of M cell, and a population of BS/m (BS enriched) cells, were prepared from 15 cm third leaves of maize B73 using the leaf rolling method of [29]; soluble proteins were extracted from each population. For each experiment, equal amounts of protein were loaded into lanes of an SDS-PAGE gel, electrophoresed, and either silver-stained or transferred to Protran nitrocellulose (Whatman) or Immobilon polyvinylidene fluoride (PVDF) membranes (EMD Millipore) for immunoblotting. Antibody reactions were detected using an ABC luminol reagent system (Amersham), and visualized using a Storm phosphorimager and ImageQuant software (GE Healthcare).

*In vivo* protein synthesis was determined by radioactively labeling 10 mm maize leaf disks placed into a solution containing 100 μCi of [^35^S]Met/Cys express labeling mix (PerkinElmer NEN Radiochemicals) in 400 μL of water. After one hour, proteins were extracted from equal wet weight of material. Tricarboxylic acid (TCA) precipitation of proteins from each extract [56], together with equal amounts (wet weight) of starting leaf material, confirmed equalized loading of samples. Protein extracts were used directly for analysis of total protein synthesis by SDS-PAGE, or immunoprecipitated from equal amounts of labeled protein extracts as described [56,95].

### RNA isolation and real-time quantitative PCR

RNA was isolated from *Arabidopsis* and maize leaves using an RNeasy Plant Mini Kit (Qiagen) according to manufactures protocols. cDNA synthesis was performed using an iScript cDNA synthesis kit (Bio-Rad) using oligo(dT) and random primers included in the kit. Real time quantative PCR (qRT-PCR) was performed using SYBR Green Supermix (Bio-Rad) on a MyiQ™2 Two Color Real-Time PCR Detection System (Bio-Rad), using primers for plastid- or nuclear-encoded mRNAs (Additional file 9) as indicated in the Figures. Quantitative expression data was normalized to *Arabidopsis* or maize *actin, rpl2, rpl20 or rps3,* as indicated in the Figures. Relative quantification in expression of the target gene relative to that of a control transcript was measured by using the 2^-ΔΔCt^ calculations. Statistical significance was calculated using Student’s T-test. For each bar shown in the graphs, P values were less than 0.05.

### RNA immunopurification with real-time quantitative PCR

Extracts for RIP/qRT-PCR were prepared from purified maize chloroplasts (containing both BS and MP plastids) as described [99] (http://pml.uoregon.edu/RIP-chip%20Protocol.pdf). 1 ml aliquots from one prepared lysate were used for each of the three immunopurification reactions described below. Each aliquot was first cleared by centrifugation at 12,000 rpm, then treated with 100 μl of Staph A cells (Pansorbin, Calbiochem), washed with CoIP buffer (20 mM Tris–HCl, pH 7.5, 150 mM NaCl 1 mM EDTA0.5% NP-405 μg/ml aprotinin for 10 minutes on ice. Three pretreated lysate aliquots were used for three types of IP reactions, using antisera against RLSB, and as controls, PEPCase and a mock reaction with no added antibody. The lysates were incubated with 15 μl of antibody (or control buffer) with rotation at 4°C overnight. The antigen-antibody complexes, and the no-antibody control reaction, were precipitated using 100 μl Staph A cells pre-treated and washed as described [95]. RNPs were disrupted by adding 25 μl 10% SDS and 10 μl 200 mM EDTA to 225 μl of the final re-suspension. 1 μl GlycoBlue (Ambion) was added to aid in recovery and visualization of small amounts of RNA. RNAs were extracted from disrupted pellets and supernatants using phenol-chloroform-isoamyl alcohol, and ethanol precipitated. The purified RNA samples were used for qRT-PCR as described above with primers for the maize chloroplast-encoded mRNAs (Additional file 9) as indicated in Figure 3, and Additional file 6: Figure S6. All data shown represent at least two repeat reactions of three independent experiments.

## Abbreviations

BS: Bundle sheath; LSU: Large subunit of Rubisco; M: Mesophyll; NAD-ME: NAD dependent malic enzyme; NADP-ME: NADP dependent malic enzyme; PEPCase: Phosphoenolpyruvate carboxylase; PPR: Pentatricopeptide repeat; PSI(II): Photosystem I(II); RLSB: *rbc*L RNA S1-binding domain protein; Rubisco: Ribulose-1,5-bisphosphate carboxylase/oxygenase; RuBP: Ribulose bisphosphate; SSU: Small subunit of Rubisco.

## Competing interest

The authors declare that they have no competing interests.

## Authors’ contributions

SMB, MP, and JOB designed and oversaw the research. SMB, MP, PY, CMM, AMZ, and JOB performed the research. CM and JAB performed bioinformatics analysis, including database searches and sequence alignments. SMB, MP, PY, CMM, AMZ, and JOB wrote the article. All authors read and approved the final manuscript.

## Supplementary Material

Additional file 1: Figure S1 RLSB proteins are present and highly conserved in a broad range of plants, including dicots, monocots, C_3_ and C_4_ species. Overall similarity among the plant species examined ranges from 50% (for maize-*Arabidopsis*), 70% (for maize-rice), to 90% (for maize-sorghum); all of these proteins have putative plastid-targeting sequences. RLSB appears to be encoded from a single copy gene in all of the species examined. This alignment used RLSB protein sequences from the following plants: maize (*Zea mays*, C_4_ monocot), accession # JX650053 (translated mRNA to protein), sorghum (*S. bicolor*, C_4_ monocot), accession # AK322408.1 (translated mRNA to protein), rice (*Oryza sativa*, C_3_ monocot), accession # NP_001043440.1, *Arabidopsis* (*A. thaliana*, C_3_ dicot), accession # JX843767 (translated mRNA to protein), tomato (*Solanum lycopersicum*, C_3_ dicot), accession # AK322408.1 (translated mRNA to protein), grape (*Vitis vinifera*, C_3_ dicot), accession # XP_002263508.1, castor bean (*Ricinus communis*, C_3_ dicot), accession # XP_002527086.1, Bienertia (*B. sinuspersici,* single cell-type C_4_ dicot) Supplied by Dr. Edwards lab, barley (*Hordeum vulgare*, C_3_ dicot), accession # BAJ92840.1, Populus (*P. tremula*, C_3_ dicot), accession # XM_002302121.1, lettuce (*Lactuca sativa,* C_3_ dicot), accession # JI580338.1. Translation of mRNA and determination of ORFs completed by using http://web.expasy.org/translate/. Multiple Protein Alignments determined on http://www.ibi.vu.nl/programs/pralinewww/. Click here for file

Additional file 2: Figure S2 Comparison of maize (accession # JX650053) and *Arabidopsis* (accession # JX843767, translated mRNA to protein) *RLSB* orthologs. Length: 345 (includes plastid transit sequences), Identity: 208/345 (60.3%), Similarity: 257/345 (74.5%). Protein Alignments determined on http://www.ibi.vu.nl/programs/pralinewww/. Click here for file

Additional file 3: Figure S3 Genomic sequence of maize *RBCL RNA S1-BINDING DOMAIN PROTIEN (RLSB)* gene (GRMZM2G087628, from Maize Genomic BAC AC211368.4), with Mu inserts. Green = Exon coding regions, Blue = introns, Black = non-coding or non-transcribed sequences, *^1^ = rblmsb1 insert; *^2^ = rblmsb2 insert. ATG and TAA are in bold and underlined. Click here for file

Additional file 4: Figure S4 Top panels: Mu-insertion mutants within the *RLSB* gene of maize cause a virescent (pale) yellow phenotype in maize seedlings. (Left top panels) Non-mutant *RLSB/RLSB* seedlings. (Right top panels) Double insertion-mutant *rlsb-1*/*rlsb-2* seedlings. Sizes in mm indicate the length of the fist leaf on a mutant plant. Note that each image pair is shown at a different scale. Bottom panels: Wild type Col0 *Arabidopsis* plants growing on MS media supplemented with increased sucrose. A stepwise increases of 3% (left) to 8% (right) sucrose was necessary to initiate and support the growth of the low Rubisco *rlsb*-silenced plants, but had no visible effects on the non-transformed Col0. Note that the plants shown in this figure are six weeks old, slightly younger than the two *rlsb*-silenced plants shown in Figure 8. Click here for file

Additional file 5: Figure S5 Long digital exposures (using ImageQuant Software) of selected western blots. Top: RLSB immunoblot blot of Figure 5A, middle panel. This enhanced, longer exposure image shows very low levels of RLSB protein accumulating in two of the *rlsb-1*/*rlsb-2* insertion mutants. Note that the lower level of RLSB in the second double mutant (lane 4), relative to the first double mutant (lane 2) corresponds to a lower level of Rubisco LSU in the same mutant, shown in the corresponding LSU lane of Figure 5, panel A. Bottom: Immunoblot of LSU Figure 8, panel C, LSU. This digitally enhanced exposure image shows that very low levels of LSU protein accumulation in two silenced *Arabidopsis* plants (S1 silenced). This digitally enhanced exposure image shows that very low levels of LSU protein accumulation in two silenced *Arabidopsis* plants (S1 silenced). Click here for file

Additional file 6: Figure S6 Accumulation of plastid-encoded mRNA and rRNA in lower leaf regions from *RLSB/RLSB* and *rlsb-1/rlsb-*2 maize seedlings. A. Accumulation of several plastid-encoded mRNAs. B. Accumulation of plastid-encoded rRNAs. C. Formaldehyde-agarose gel, transferred to nitrocellulose and stained with methylene blue, showing cytoplasmic and chloroplast rRNAs in two RLSB/RLSB plants and three *rlsb-1/rlsb-2* mutant plants. 28S and 18S are cytoplasmic rRNAs. 16S rRNA and the 23S* cleavage product of 23S rRNA are chloroplastic. For qRT-PCR shown in A and B, quantification of transcript levels was standardized to actin mRNA. Data is averaged for two RLSB/RLSB plants and four *rlsb-1/rlsb-2* siblings, with three repeats run for each of the plant samples. Note differences in scale for panels A and B, due to the much greater abundance of rRNA relative to the mRNAs. Statistical significance was calculated using Student’s t-test. For each bar, P values were less than 0.05. Click here for file

Additional file 7: Figure S7 Low magnification immunolocalization image of RLSB and Rubisco LSU proteins in leaf sections of the C_3_ plant *Arabidopsis*. A. *Arabidopsis* leaf section reacted with RLSB primary antiserum. B. *Arabidopsis* leaf sections reacted with LSU primary antiserum. C. *Arabidopsis* leaf section showing autofluorescence of plastids (imaged enhanced) from a section reacted with secondary antibody alone. D. DIC image of the images shown in A and C, with chloroplasts indicated. cp, chloroplasts. *Arabidopsis* leaf sections were incubated with the indicated primary antiserum, and then with R-phycoerythrin (A, B) conjugated secondary antibody. Images were captured using a 20X objective of a Leica DMIRE2 inverted fluorescent microscope, bar = 100 μM. Click here for file

Additional file 8: Figure S8 Rubisco protein and mRNA accumulation in *rlsb* T-DNA insertion heterozygotes, non-mutant siblings, and wild type Col0 *Arabidopsis*. A. Western analysis. Total protein from heterozygous SALK_015722 containing a T-DNA insert in one copy of the *RLSB* locus, and wild type non-insert containing plants reacted with LSU antisera (top panels). Equal amounts of protein were loaded in each lane. As a control, the blot was re-probed with actin antisera (bottom panels). B. Segregating wild type siblings lacking T-DNA inserts showed no LSU reduction. C. qRT-PCR analysis of *rbc*L mRNA in wild type and SALK_015722 heterozygotes. For each plant, random primers produced cDNA from mRNA; these were then incubated with primers for *Arabidopsis rbc*L mRNAs, or for plastid-encoded *rps3* and *rpl20* (standardization controls). For each bar, P values were less than 0.05. D. Genomic PCR analysis of T-DNA insert in At1g71720 locus. Ethidium-bromide stained agarose gel showing representative PCR amplifications using total DNA isolated from the indicated plants. The three primers added to each PCR reaction were: LP (At1g71720 sequence upstream of insert site) = TCGATTGCTGATTTTGATTCC; RP (At1g71720 sequence downstream of insert site) = TTCCTTCCCCTTTTTCATGTC; LBB1 (left border of T-DNA) = GCGTGGACCGCTTGCTGCAACT (Reverse AGTTGCAGCAAGCGGTCCACGC). Sequencing confirmed the identity of the amplified fragments. A 261 nucleotide band corresponding to wild type At1g71720 was amplified from LP and RP primers if there was no insert; A 128 nt band was amplified from LP and LBB1 if the locus contained an insert. Lane 1 = wt Col0 (normal LSU levels), showing a band of 261 nt. Lane 2 = heterozygote AT SALK 017226 (reduced LSU), showing two amplified bands of 261 and 128 nt. Lane 3 = AT SALK 017226 segregate (sibling plant with normal LSU levels and no T-DNA insert), showing a single amplified band at 261 nt. Click here for file

Additional file 9 List of primer sequences used for this study. Click here for file

## References

[B1] Raghavendra AS Sage RF Raghavendra AS, Sage RF Introduction C4 Photosynthesis and Related CO2 Concentrating Mechanisms. Advances in Photosynthesis and Respiration, Volume 32 2011 The Netherlands: Springer Dordrecht 17 25

[B2] Sage RF Zhu X-G Exploiting the engine of C_4_ photosynthesis J Exp Bot 2011 62 2989 3000 10.1093/jxb/err179 21652533

[B3] Sage RF Sage TL Kocacinar F Photorespiration and the evolution of C_4_ photosynthesis Ann Rev Plant Biol 2012 63 19 47 10.1146/annurev-arplant-042811-105511 22404472

[B4] Jones MB Raghavendra AS, Sage RF C_4_ species as energy crops C4 Photosynthesis and Related CO2 Concentrating Mechanisms. Advances in Photosynthesis and Respiration, Volume 32 2011 The Netherlands: Springer Dordrecht 379 397

[B5] Berry JO Zielinski AM Patel M Raghavendra AS, Sage RF Gene expression in mesophyll and bundle sheath cells of C_4_ plants C4 Photosynthesis and Related CO2 Concentrating Mechanisms. Advances in Photosynthesis and Respiration, Volume 32 2011 The Netherlands: Springer Dordrecht 221 256

[B6] Berry JO Yerramsetty P Zielinski AM Mure C Photosynthetic gene expression in higher plants Photosyn Res 2013 doi:10.1007/s11120-013-9880-8 10.1007/s11120-013-9880-8 23839301

[B7] Patel M Berry JO Rubisco gene expression in C_4_ plants J Exp Bot 2008 59 1625 1634 18325924 10.1093/jxb/erm368

[B8] von Caemmerer S Furbank RT The C_4_ pathway: an efficient CO_2_ pump Photosyn Res 2003 77 191 207 10.1023/A:1025830019591 16228376

[B9] Hatch MD C_4_ photosynthesis: a unique blend of modified biochemistry, anatomy, and ultrastructure Biochem Biophys Acta 1987 895 81 106 10.1016/S0304-4173(87)80009-5

[B10] Edwards GE Voznesenskaya EV Raghavendra AS, Sage RF C_4_ photosynthesis: Kranz forms and single-cell C_4_ in terretrial plants C4 Photosynthesis and Related CO2 Concentrating Mechanisms. Advances in Photosynthesis and Respiration, Volume 32 2011 The Netherlands: Springer Dordrecht 29 61

[B11] Hibberd JM Covshoff S The regulation of gene expression required for C_4_ photosynthesis Ann Rev Plant Biol 2010 61 181 207 10.1146/annurev-arplant-042809-112238 20192753

[B12] Langdale J C_4_ Cycles: past, present, and future research on C_4_ photosynthesis Plant Cell 2011 23 3879 3892 10.1105/tpc.111.092098 22128120 PMC3246324

[B13] Tyagi AK Gaur T Light regulation of nuclear photosynthetic genes in higher plants Crit Rev Plant Sci 2003 22 417 452

[B14] Raynaud C Loisela C Wostrikoff K Kuras R Girard-Bascou J Wollman FA Choquet Y Evidence for regulatory function of nucleus-encoded factors on mRNA stabilization and translation in the chloroplast Proc Natl Acad Sci U S A 2007 104 9093 9098 10.1073/pnas.0703162104 17494733 PMC1885633

[B15] Johnson X Wostrikoff K Finazzi G Kuras R Schwarz C Bujaldon S Nickelsen J Stern DB Wollman F-A Vallon O MRL1, a conserved pentatricopeptide repeat protein, is required for stabilization of *rbc*L mRNA in *Chlamydomonas* and *Arabidopsis* Plant Cell 2010 22 234 248 10.1105/tpc.109.066266 20097872 PMC2828700

[B16] Tillich M Beick S Schmitz-Linneweber C Chloroplast RNA-binding proteins: repair and regulation of chloroplast transcripts RNA Biol 2010 7 172 178 10.4161/rna.7.2.11090 20215878

[B17] Barkan A Expression of plastid genes: organelle-specific elaborations on a prokaryotic scaffold Plant Physiol 2011 155 1520 1532 10.1104/pp.110.171231 21346173 PMC3091090

[B18] Slewinski TL Anderson AA Zhang C Turgeon R Scarecrow plays a role in establishing kranz anatomy in maize leaves Plant Cell Physiol 2013 53 2030 2037 23128603 10.1093/pcp/pcs147

[B19] Gutteridge S Gatenby AA Rubisco synthesis, assembly, mechanism, and regulation Plant Cell 1995 7 809 819 12242387 10.1105/tpc.7.7.809 PMC160870

[B20] Rodermel S Subunit control of Rubisco biosynthesis - a relic of an endosymbiotic past? Photosyn Res 1999 59 105 123 10.1023/A:1006122619851

[B21] Wostrikoff K Clark A Sato S Clemente T Stern D Ectopic expression of rubisco subunits in maize mesophyll cells does not overcome barriers to cell type-specific accumulation Plant Physiol 2012 160 419 432 10.1104/pp.112.195677 22744982 PMC3440216

[B22] Brown NJ Newell CA Stanley S Chen JE Perrin AJ Kajala K Hibberd JM Independent and parallel recruitment of pre-existing mechanisms underlying C_4_ photosynthesis Science 2011 331 1436 1439 10.1126/science.1201248 21415351

[B23] Manuell A Beligni MV Yamaguchi K Mayfield SP Regulation of chloroplast translation: interactions of RNA elements, RNA-binding proteins and the plastid ribosome Biochem Soc Trans 2004 32 601 605 10.1042/BST0320601 15270686

[B24] Stern DB Goldschmidt-Clermont M Hanson MR Chloroplast RNA metabolism Annu Rev Plant Biol 2010 61 125 155 10.1146/annurev-arplant-042809-112242 20192740

[B25] Schmitz-Linneweber C Small I Pentatricopeptide repeat proteins: a socket set for organelle gene expression Trends Plant Sci 2008 13 663 670 10.1016/j.tplants.2008.10.001 19004664

[B26] McCormac DJ Litz H Wang J Gollnick PD Berry JO Light-associated and processing-dependent protein binding to the 5′ UTR of *rbc*L mRNA in the chloroplasts of a C_4_ plant J Biol Chem 2001 276 3476 3483 10.1074/jbc.M009236200 11076953

[B27] Yao W Muqtadir K Bruenn JA Packaging in a yeast double-stranded RNA virus J Virol 1995 69 1917 1919 7853534 10.1128/jvi.69.3.1917-1919.1995 PMC188807

[B28] Maai E Miyake H Taniguchi M Differential positioning of chloroplasts in C_4_ mesophyll and bundle sheath cells Plant Signal Behav 2011 6 111 113 10.4161/psb.6.8.15809 PMC3260704 21757999

[B29] Covshoff S Furbank RT Leegood RC Hibberd JM Leaf rolling allows quantification of mRNA abundance in mesophyll cells of sorghum J Exp Bot 2012 64 807 813 23077203 10.1093/jxb/ers286

[B30] Li P Ponnala L Gandotra N Wang L Si Y Tausta SL Kebrom TH Provart N Patel R Myers CR Reidel EJ Turgeion R Liu P Sun Q Nelson T Brutnell TP The developmental dynamics of the maize leaf transcriptome Nature Genet 2010 42 1060 1067 10.1038/ng.703 21037569

[B31] Majeran W Friso G Ponnala L Connolly B Huang M Reidel E Zhang C Asakura Y Bhuiyan NH Sun Q Turgeon R Wijk KJ Structural and metabolic transitions of C_4_ leaf development and differentiation defined by microscopy and quantitative proteomics in maize Plant Cell 2010 22 3509 3542 10.1105/tpc.110.079764 21081695 PMC3015116

[B32] Schnable PS Ware D Fulton RS Stein JC Wei F Pasternak S The B73 maize genome: complexity, diversity, and dynamics Science 2009 326 1112 1115 10.1126/science.1178534 19965430

[B33] Chang YM Liu WY Shih AC Shen MN Lu CH Lu MY Yang HW Wang TY Chen SC Chen SM Li WH Ku MS Characterizing regulatory and functional differentiation between maize mesophyll and bundle sheath cells by transcriptomic analysis Plant Physiol 2002 160 165 177 22829318 10.1104/pp.112.203810 PMC3440195

[B34] Khalil AM Guttman M Huarte M Garber M Raj A Morales DR Thomas K Presser A Bernstein BE van Oudenaarden A Regev A Lander ES Rinn JL Many human large intergenic noncoding RNAs associate with chromatin-modifying complexes and affect gene expression Proc Natl Acad Sci U S A 2009 106 11667 11672 10.1073/pnas.0904715106 19571010 PMC2704857

[B35] Selth LA Gilbert C Svejstrup JQ RNA immunoprecipitation to determine RNA-protein associations *in vivo* Cold Spring Harb Protoc 2009 pdb.prot5234 doi:10.1101/pdb.prot5234 20147192 10.1101/pdb.prot5234

[B36] Steeves TA Sussex IM Patterns in Plant Development 1989 Cambridge: Cambridge University Press

[B37] Sylvester AW Cande WZ Freeling M Division and differentiation during normal and liguleless-1 maize leaf development Development 1990 110 985 1000 2088734 10.1242/dev.110.3.985

[B38] Barkan A Nuclear mutants of maize with defects in chloroplast polysome assembly have altered chloroplast RNA metabolism Plant Cell 1993 5 389 402 12271069 10.1105/tpc.5.4.389 PMC160279

[B39] Sheen J-Y Bogorad L Differential expression of genes for photosystem II components encoded by the plastid genome in bundle sheath and mesophyll cells of maize Plant Physiol 1988 86 1020 1026 10.1104/pp.86.4.1020 16666025 PMC1054621

[B40] Li Y Sugiura M Three distinct ribonucleoproteins from tobacco chloroplasts: each contains a unique amino terminal acidic domain and two ribonucleoprotein consensus motifs EMBO J 1990 9 3059 3066 1698606 10.1002/j.1460-2075.1990.tb07502.x PMC552030

[B41] Rhee SY Beavis W Berardini TZ Chen G Dixon D Doyle A Garcia-Hernandez M Huala E Lander G Montoya M Miller N Mueller LA Mundodi S Reiser L Tacklind J Weems DC Wu Y Xu I Yoo D Yoon J Zhang P The *Arabidopsis* information resource (TAIR): a model organism database providing a centralized, curated gateway to *Arabidopsis* biology, research materials and community Nuc Acids Res 2003 31 224 228 10.1093/nar/gkg076 PMC165523 12519987

[B42] Hetherington SE Smillie RM Davies WJ Photosynthetic activities of vegetative and fruiting tissues of tomato J Exp Bot 1999 49 1173 1181

[B43] Pyke K Mesophyll eLS 2012 doi:10.1002/9780470015902.a0002081.pub2

[B44] Bycroft M Hubbard TJP Proctor M Freund SMV Murzin AG The solution structure of the S1 RNA binding domain: A member of an ancient nucleic acid–binding fold Cell 1997 88 235 242 10.1016/S0092-8674(00)81844-9 9008164

[B45] Yehudai-Resheff S Portnoy V Yogev S Adir N Schuster G Domain analysis of the chloroplast polynucleotide phosphorylase reveals discrete functions in RNA degradation, polyadenylation, and sequence homology with exosome proteins Plant Cell 2003 15 2003 2019 10.1105/tpc.013326 12953107 PMC181327

[B46] Schein A Sheffy-Leven S Glaser F Schuster G The RNase E/G-type endoribonuclease of higher plants is located in the chloroplast and cleaves RNA similarly to the *E. coli* enzyme RNA 2008 2008 14 1057 1068 18441049 10.1261/rna.907608 PMC2390796

[B47] Chekanova JA Dutko JA Mian IS Belostotsky DA Arabidopsis thaliana exosome subunit AtRrp4p is a hydrolytic 3′–>5′ exonuclease containing S1 and KH RNA-binding domains Nucl Acids Res 2002 30 695 700 10.1093/nar/30.3.695 11809881 PMC100302

[B48] Worbs M Bourenkov GP Bartunik HD Huber R Wahl MC An extended RNA binding surface through arrayed S1 and KH domains in transcription factor NusA Mol Cell 2011 7 1177 1189 11430821 10.1016/s1097-2765(01)00262-3

[B49] Stickney LM Hankins JS Miao X Mackie GA Function of the conserved S1 and KH domains in polynucleotide phosphorylase J Bacteriol 2005 187 7214 7221 10.1128/JB.187.21.7214-7221.2005 16237005 PMC1272994

[B50] Eklund EA Lee S Skalnik DG Cloning of a cDNA encoding a human DNA-binding protein similar to ribosomal protein S1 Gene 1995 155 231 235 10.1016/0378-1119(94)00898-3 7721096

[B51] Majeran W Friso G Asakura Y Qu X Huang M Ponnala L Watkins KP Barkan A van Wijk KJ Nucleoid-enriched proteomes in developing plastids and chloroplasts from maize leaves: a new conceptual framework for nucleoid functions Plant Physiol 2012 158 156 189 10.1104/pp.111.188474 22065420 PMC3252073

[B52] Romanowska E Drożak A Comparative analysis of biochemical properties of mesophyll and bundle sheath chloroplasts from various subtypes of C_4_ plants grown at moderate irradiance Acta Bioch Pol 2006 53 709 719 17106510

[B53] Simcox PD Garland W Deluca V Canvin DT Dennis DT Respiratory pathways and fat synthesis in the developing castor-oil seed Can J Bot 1979 57 1008 1014 10.1139/b79-125

[B54] Ruuska SA Girke T Benning C Ohlrogge JB Contrapuntal networks of gene expression during *Arabidopsis* seed filling Plant Cell 2002 14 1191 1206 10.1105/tpc.000877 12084821 PMC150774

[B55] Schwender J Goffman F Ohlrogge JB Shachar-Hill Y Rubisco without the Calvin cycle improves the carbon efficiency of developing green seeds Nature 2004 432 779 782 10.1038/nature03145 15592419

[B56] Berry JO Nikolau BJ Carr JP Klessig DF Transcriptional and post-transcriptional regulation of ribulose 1,5-bisphosphate carboxylase gene expression in light- and dark-grown amaranth cotyledons Mol Cell Biol 1985 5 2238 2246 3837189 10.1128/mcb.5.9.2238-2246.1985 PMC366949

[B57] Yamamoto N Mukai Y Matsuoka M Kano-Murakami Y Tanaka Y Oshashi Y Ozeki Y Odani K Light-independent expression of *cab* and *rbc*S genes in dark-grown pine seedlings Plant Physiol 1991 95 379 383 10.1104/pp.95.2.379 16667994 PMC1077541

[B58] Maeda Y Minamikawa T Xu B-M Metabolic activities in germinated ancient lotus seeds J Exp Bot 1996 47 577 582 10.1093/jxb/47.4.577

[B59] Romanowska E Kargul J Powikrowska M Finazzi G Nield J Drozak A Pokorska B Structural organization of photosynthetic apparatus in agranal chloroplasts of maize J Biol Chem 2008 283 26037 26046 10.1074/jbc.M803711200 18632664 PMC3258860

[B60] Kleine T Maier UG Leister D DNA transfer from organelles to the nucleus: the idiosyncratic genetics of endosymbiosis Annu Rev Plant Biol 2009 60 115 138 10.1146/annurev.arplant.043008.092119 19014347

[B61] Zhelyazkova P Sharma CM Förstner KU Liere K Vogel J Börner T The primary transcriptome of barley chloroplasts: numerous noncoding RNAs and the dominating role of the plastid-encoded RNA polymerase Plant Cell 2012 2012 24 123 136 22267485 10.1105/tpc.111.089441 PMC3289561

[B62] Mullet JE Klein RR Transcription and RNA stability are important determinants of higher plant chloroplast RNA levels EMB0 J 1987 6 1571 1579 10.1002/j.1460-2075.1987.tb02402.x PMC553526 16453773

[B63] Whitney SM Andrews TJ The gene for the ribulose-1,5-bisphosphate carboxylase/oxygenase (Rubisco) small subunit relocated to the plastid genome of tobacco directs the synthesis of small subunits that assemble into Rubisco Plant Cell 2001 13 193 205 11158539 10.1105/tpc.13.1.193 PMC102209

[B64] Chalker-Scott L Environmental significance of anthocyanins in plant stress responses Photochem Photobiol 1999 70 1 9 10.1111/j.1751-1097.1999.tb01944.x

[B65] Solfanelli C Poggi A Loreti E Alpi A Perata P Sucrose-specific induction of the anthocyanin biosynthetic pathway in *Arabidopsis* Plant Physiol 2006 140 637 646 10.1104/pp.105.072579 16384906 PMC1361330

[B66] Long JJ Berry JO Tissue-specific and light-mediated expression of the C_4_ photosynthetic NAD-dependent malic enzyme of amaranth mitochondria Plant Physiol 1996 112 473 482 12226404 10.1104/pp.112.2.473 PMC157970

[B67] Bowman SM Drzewiecki KD Mojica E-R Zielinski AM Siegel A Aga DA Berry JO Toxicity and reductions in intracellular calcium levels following?uptake of a tetracycline antibiotic in *Arabidopsis* Environ Sci&Technol 2011 45 8958 8964 10.1021/es200863j 21882870

[B68] Rodermel SR Abbott MS Bogorad L Nuclear-organelle interactions: nuclear antisense gene inhibits ribulose bisphosphate carboxylase enzyme levels in transformed tobacco plants Cell 1988 55 673 681 10.1016/0092-8674(88)90226-7 3052855

[B69] Whitney SM von Caemmerer S Hudson GS Andrews TJ Directed mutation of the rubisco large subunit of tobacco influences photorespiration and growth Plant Physiol 1999 121 579 588 10.1104/pp.121.2.579 10517850 PMC59421

[B70] Dhingra A Portis AR Jr Daniell H Enhanced translation of a chloroplast-expressed *Rbc*S gene restores small subunit levels and photosynthesis in nuclear RbcS antisense plants Proc Natl Acad Sci U S A 2004 101 6315 6320 10.1073/pnas.0400981101 15067115 PMC395966

[B71] Ichikawa K Miyake C Iwano M Sekine M Shinmyo A Kato K Ribulose 1,5-bisphosphate carboxylase/oxygenase large subunit translation is regulated in a small subunit-independent manner in the expanded leaves of tobacco Plant Cell Physiol 2008 49 214 225 18178584 10.1093/pcp/pcm179

[B72] Feiz L Williams-Carrier R Wostrikoff K Belcher S Barkan A Stern DB Ribulose-1,5-bis-phosphate carboxylase/oxygenase accumulation factor1 is required for holoenzyme assembly in maize Plant Cell 2012 24 3435 3466 10.1105/tpc.112.102012 22942379 PMC3462642

[B73] Foyer CH Neukermans J Queval G Noctor G Harbinson J Photosynthetic control of electron transport and the regulation of gene expression J Exp Bot 2012 63 1637 1661 10.1093/jxb/ers013 22371324

[B74] Barnes D Mayfield SP Redox control of posttranscriptional processes in the chloroplast Antiox Redox Sig 2003 5 89 94 10.1089/152308603321223577 12626120

[B75] Raghavendra AS Padmasre: beneficial interactions of mitochondrial metabolism with photosynthetic carbon assimilation Trends Plant Sci 2003 8 546 553 10.1016/j.tplants.2003.09.015 14607100

[B76] Puthiyaveetil S Ibrahim IM Allen JF Oxidation–reduction signalling components in regulatory pathways of state transitions and photosystem stoichiometry adjustment in chloroplasts Plant Cell Environ 2012 35 347 359 10.1111/j.1365-3040.2011.02349.x 21554328

[B77] Rossel JB Wilson IW Pogson BJ Global changes in gene expression in response to high light in *Arabidopsis* Plant Physiol 2002 130 1109 1120 10.1104/pp.005595 12427978 PMC166632

[B78] Liu M-J Wu S-H Chen H-M Wu S-H Widespread translational control contributes to the regulation of *Arabidopsis* photomorphogenesis Mol Sys Biol 2012 8 566 10.1038/msb.2011.97 PMC3296358 22252389

[B79] Park SH Chung PJ Juntawong P Bailey-Serres J Kim YS Jung H Bang SW Kim YK Do Choi Y Kim JK Posttranscriptional control of photosynthetic mRNA decay under stress conditions requires 3′ and 5′ untranslated regions and correlates with differential polysome association in rice Plant Physiol 2012 159 1111 1124 10.1104/pp.112.194928 22566494 PMC3387698

[B80] Paul MJ Foyer CH Sink regulation of photosynthesis J Exp Bot 2001 52 1383 1400 10.1093/jexbot/52.360.1383 11457898

[B81] Maier A Zell MB Maurino VG Malate decarboxylases: evolution and roles of NAD(P)-ME isoforms in species performing C_4_ and C_3_ photosynthesis J Exp Bot 2010 62 3061 3069 21459769 10.1093/jxb/err024

[B82] Weissmann S Brutnell TP Engineering C_4_ photosynthetic regulatory networks Curr Opin Biotechnol 2012 23 298 304 10.1016/j.copbio.2011.12.018 22261559

[B83] Lee KP Kim C Landgraf F Apel K EXECUTER1- and EXECUTER2-dependent transfer of stress-related signals from the plastid to the nucleus of *Arabidopsis thaliana* Proc Natl Acad Sci U S A 2007 104 10270 10275 10.1073/pnas.0702061104 17540731 PMC1891253

[B84] Slesak I Libik M Karpinska B Karpinski S Miszalski Z The role of hydrogen peroxide in regulation of plant metabolism and cellular signaling in response to environmental stresses Acta Biochim Pol 2007 54 39 50 17325747

[B85] Piippo M Allahverdiyeva Y Paakkarinen V Suoranta UM Battchikova N Aro EM Chloroplast-mediated regulation of nuclear genes in *Arabidopsis thaliana* in the absence of light stress Physiol Genom 2006 25 142 152 10.1152/physiolgenomics.00256.2005 16403842

[B86] Acevedo-Hernandez GJ Leon P Herrera-Estrella LR Sugar and ABA responsiveness of a minimal RBCS light-responsive unit is mediated by direct binding of ABI4 Plant J 2005 43 506 519 10.1111/j.1365-313X.2005.02468.x 16098105

[B87] Langdale JA Kidner CA Bundle sheath defective, a mutation that disrupts cellular differentiation in maize leaves Development 1994 120 673 681

[B88] May BP Martienssen RA Transposon mutagenesis in the study of plant development Crit Rev Plant Sci 2003 22 1 35 10.1080/713610849

[B89] Lisch D Mutator transposons Trends Plant Sci 2002 11 498 504 12417150 10.1016/s1360-1385(02)02347-6

[B90] Barkan A Martienssen RA Inactivation of maize transposon Mu suppresses a mutant phenotype by activating an outward-reading promoter near the end of Mu1 Proc Natl Acad Sci U S A 1991 88 3502 3506 10.1073/pnas.88.8.3502 1849660 PMC51476

[B91] Ortiz DF Strommer JN The Mu1 maize transposable element induces tissue-specific aberrant splicing and polyadenylation in two Adh1 mutants Mol Cell Biol 1990 10 2090 2095 2157968 10.1128/mcb.10.5.2090-2095.1990 PMC360556

[B92] Hudson GS Mahon JD Anderson PA Gibbs MJ Badger MR Andrews T Whitfeld PR Comparisons of *rbc*L genes for the large subunit of ribulose-bisphosphate carboxylase from closely related C_3_ and C_4_ plant species J Biol Chem 1990 265 808 814 2295620

[B93] Patel M Corey AC Yin L-Y Ali S Taylor WC Berry JO Untranslated regions from C_4_ amaranth *AhRbc*S1 mRNAs confer translational enhancement and preferential bundle sheath cell expression in transgenic C_4_*Flaveria bidentis* Plant Physiol 2004 136 3550 3561 10.1104/pp.104.051508 15489276 PMC527154

[B94] Wang J-L Klessig DF Berry JO Regulation of C_4_ gene expression in developing amaranth leaves Plant Cell 1992 4 173 184 12297645 10.1105/tpc.4.2.173 PMC160118

[B95] McCormac DJ Boinski JJ Ramsperger VC Berry JO C_4_ Gene expression in photosynthetic and non-photosynthetic leaf regions of *Amaranthus tricolor* Plant Physiol 1997 114 801 815 12223743 10.1104/pp.114.3.801 PMC158366

[B96] Wesley SV Helliwell CA Smith NA Wang NB Rouse DT Liu Q Gooding PS Singh SP Abbott D Stoutjesdijk PA Robinson SP Gleave AP Green AG Waterhouse PM Construct design for efficient, effective and high-throughput gene silencing in plants Plant J 2001 27 581 590 10.1046/j.1365-313X.2001.01105.x 11576441

[B97] Bent A *Arabidopsis thaliana* floral dip transformation method Methods Mol Biol 2006 343 87 103 16988336 10.1385/1-59745-130-4:87

[B98] Barkan A Approaches to investigating nuclear genes that function in chloroplast biogenesis in land plants Meth Enzymol 1998 297 38 57

[B99] Barkan A Genome-wide analysis of RNA-protein interactions in plants Meth Mol Biol 2009 553 13 37 10.1007/978-1-60327-563-7_2 19588099

