# Supplementary material for: A novel RNA binding protein affects rbcL gene expression and is specific to bundle sheath chloroplasts in C4 plants
Source: BMC Plant Biol. 2013 Sep 22;13:138. doi: 10.1186/1471-2229-13-138 (PMC3849040; doi:10.1186/1471-2229-13-138)
Supplement: Additional file 1: Figure S1 — RLSB proteins are present and highly conserved in a broad range of plants, including dicots, monocots, C3 and C4 species. Overall similarity among the plant species examined ranges from 50% (for maize-Arabidopsis), 70% (for maize-rice), to 90% (for maize-sorghum); all of these proteins have putative plastid-targeting sequences. RLSB appears to be encoded from a single copy gene in all of the species examined. This alignment used RLSB protein sequences from the following plants: maize (Zea mays, C4 monocot), accession # JX650053 (translated mRNA to protein), sorghum (S. bicolor, C4 monocot), accession # AK322408.1 (translated mRNA to protein), rice (Oryza sativa, C3 monocot), accession # NP_001043440.1, Arabidopsis (A. thaliana, C3 dicot), accession # JX843767 (translated mRNA to protein), tomato (Solanum lycopersicum, C3 dicot), accession # AK322408.1 (translated mRNA to protein), grape (Vitis vinifera, C3 dicot), accession # XP_002263508.1, castor bean (Ricinus communis, C3 dicot), accession # XP_002527086.1, Bienertia (B. sinuspersici, single cell-type C4 dicot) Supplied by Dr. Edwards lab, barley (Hordeum vulgare, C3 dicot), accession # BAJ92840.1, Populus (P. tremula, C3 dicot), accession # XM_002302121.1, lettuce (Lactuca sativa, C3 dicot), accession # JI580338.1. Translation of mRNA and determination of ORFs completed by using http://web.expasy.org/translate/. Multiple Protein Alignments determined on http://www.ibi.vu.nl/programs/pralinewww/. [file 1471-2229-13-138-S1.pdf]

Additional File 1: Figure S1

Results colour-coded for amino acid conservation

The current colourscheme of the alignment is for **amino acid conservation**.

The conservation scoring is performed by PRALINE. The scoring scheme works from 0 for the least conserved alignment position, up to 10 for the most conserved alignment position. The colour assignments are:

Unconserved 0 1 2 3 4 5 6 7 8 9 10 **Conserved**

```
            .........10 .........20 .........30 .........40 .........50
Maize       ---------- ---------- ---------- ---------- ----------
Sorghum     ---------- ---------- ---------- ---------- ----------
Arabidopsis MQTLLCQ--P -CKSLPILT- ASSSSSLIRS SGDVRECIDF RASEKVSKFQ
Tomato      MP-LLLL--P -CKSFSIFN- PILPLNTSVI YNTAIQFSGF SLS-----PK
Bienertia   MPVVM----- --KSHSPLS- VLSSFNPAVP STPYHLCK-- NAN-------
Grape       MPIVLAH--P -SNSLSF--- RFSSHPPLNS NISFTS---F HNT-----PK
Rice        ---------- ---------- ---------- ---------- ----------
Castor      ----M----R -CK-LNRI-- ---------- ---------- ------TKDA
Barley      ----MPP--P -SL-LSLLP- AASLSPRTRR ---------- ------PKRR
Populus     MQILLLQ--P -CKSLTLFSY SLPSNTDSLV ITHKRSIVHH NKCAKPPCFH
Lettuce     MSLLLLHHHP CCKFLPPLDT FITCKTPINR YISCPKTPKP FNN--LPFLT
Consistency 2112410002 0232321210 1121111111 1010010000 1110000001

            .........60 .........70 .........80 .........90 .........100
Maize       ---------- ---------- -MPTPAPALP FV----PKPQ PILHVAKALV
Sorghum     ---------- ---------- -MPAPAPPLP SL----PKPQ PILHVAKALV
Arabidopsis FHVTLSPFAF -RGFSICREF AVRGAYGIRF CS----REDV SGVGNGGIVA
Tomato      YPLPRTPKSS -KNLSIHWNY QINLPTHVLF CS----KNEI FEEFRTTQLA
Bienertia   -RILLKKLLF PQNLGFCGNY SIWKASHVSF CY----AKDK D-ISEGFPE-
Grape       LPLRKPQSKT --LVSPKNSP VWR-TTQISF CSPNDIFYDI SSTQLPETPE
Rice        ---------- ---------- -------MPP SM----AVAA AAAAPRSLLF
Castor      NPFSALSTQI -SFCSQNDTF DNL-SSTQVP EE----QEED RIRENEELEL
Barley      APAANHRLRC -RVPVIVSAR APS-PLRSLP SS----SDVV EGGVGGEGGE
Populus     SFCSLGTL-- -KAIFLSKNS QLWKSSLVTL CSQNDVFDDF SSTQLPEKER
Lettuce     KYSICRNTHV -VNCSNNEVI DEFTNTQLDQ TQ----ENEI EELGLLGKPS
Consistency 1121112211 0111211121 2211342433 4400003443 3333334333

            .........110.........120.........130.........140.........150
Maize       P--SPRQTQP ---------- ---------- -------QAR GRGLPQPVNC
Sorghum     L--SPRQAQP ---------- ---------- -------QAR GRGRPQPVMC
Arabidopsis E------EEI ELLNK----- --PNPLPKSE N------EES GKADDDAILE
Tomato      E--LPESEEL ELHNK----- --PYLKQIDN GVVSDVEEDQ KKVSKDEVLE
Bienertia   ----IEKLAD SEELE----- --LLDKPSPM PVNGDTDTLE NEPKRPDKNA
Grape       I--DGVQDIE ELELLGKPSP VPLNNGSASD IDSELK-KPD KDEALAPFLK
Rice        P--VSPSPSQ PRRV------ -------PAL ARAG---R-- ---SQRRHAA
Castor      L--NKPSPVV INNGL----- --SIEVDKES ETID---KDE ALAPFLKFFE
Barley      E--LHLLDKP FPSPS----- --APEE--DE PEPE---AAP APSTADALAP
Populus     DDRIQVNEEL ELLNKPSPVI FNNGLDVEAD KESEKPGKDE ALAPFLKFFK
Lettuce     P--MPVINVP PQVES----- ---------- -------EYI KPQKEEVLEP
Consistency 2002223343 2222200000 0010110122 1111000523 3233233433

            .........160.........170.........180.........190.........200
Maize       ANSKRLDDAL SAGFVRLLNA GQVQDADSAS GTCRRYDP-- --KPGDFAVG
Sorghum     ASSKRLDDAL SAGFVRLLNA GHVQDADSAS GTDRRYDP-- --KPGDFAVG
Arabidopsis PFLKFFKPE- -----EEGEG IESEVSD--E TDRVSVEY-Y DPKPGDFVVG
Tomato      PFYKLFKPIE SNEEESDIEQ EEEVHPVVEE SKKVSVEY-Y EPKPGDLVVG
Bienertia   ALEPFLKFFR TTDMEEEVEP AEQVVAKLEE ESDKVIVEYY EPKPGDFVVG
Grape       FFKPRESSEE ANGASGEDGS EISESGSTKL VSVEYYEP-- --KPGDFVVG
Rice        LARPARRHLD --DAMSAG-F VRSLNAPPDE DGARSGGR-Y DPRPGDFAVG
Castor      PRDSLEEIKE EGKELGVIEG NSNGNNEDKE AKKVNVDY-Y EPKPGDFVVG
Barley      YLNFFQVKGA EADVAAT--A GKDSSEGGPG TGGRGVIY-F DPKPGDLVAG
Populus     SNDSLDEVSE DERDLGVVEE RSGVDNEDKE PRKINVDY-Y EPKPGDFVVG
Lettuce     FYKFFRDGKS AEETSDSEDV SEEISMEEEE DKKVSVEY-Y DPKQGDFVVG
Consistency 3334434233 3232344233 3443444245 3434454404 4598**879*

            .........210.........220.........230.........240.........250
Maize       VVVSGTEARL DVAVGADRLA TLLSKELLPL DRDSSDPP-- --------TR
Sorghum     VVVSGTEARL DVAVGADRLA TLLAKELLPL DRDSADSP-- --------TR
Arabidopsis VVVSGNENKL DVNIGADMLG TMLTKEILPL YDKELDYLLC DLK----YDA
Tomato      VVVSGNENKL DVSIGADLLG TMLTKDVLPL YDKEIGYLLC DLE----KDA
Bienertia   VVVSGNENKL DVNIGADLLG TMLTKEVLPL YDKEMDYMLR DLD----KNA
Grape       VVVSGNENKL DVNVGADLLG TMLTKEVLPL YDKEMEYLLC DVE----KDA
Rice        VVVSGTEARL DVAVGADRLA TLLAKELLPL YRADLPAFAE AAPPRPGSVG
Castor      VVVSGNESKL DVNVGADLLG TMLTKEVLPL YDKEMEYLLC --DME-KDAE
```

```
Barley      VVVGGDARAL DVDVGAGGEP ALMLTKEAAP ASPEEFAYLA CDVAS-GGAR
Populus     VVVSGNENKL DVNIGAD--- ---------- ---------- ----------
Lettuce     VVVSGNEYKL DVNIGADLLG TMLTKEVLPL YEKELDNLLC DFE----KNP
Consistency ***8*6847* **59**8365 7675765776 4446443342 2120000233

            .........260.........270.........280.........290.........300
Maize       QALPRLGSIG VVAGPSVD-- --DDRQKRGS RTLVAPGTVV FAEVLGRTLS
Sorghum     QALPGPGSIG VVAGLTVDDD KEARRQKRGS RTLVAPGTVV FAEVLGRTLS
Arabidopsis EEFLVNGKMG IVKDDDEGV- -EIAEFARQG RPVVEIGTVV FAEVLGRTLS
Tomato      EEFLVRGKMG ILSYDDAVS- ----GESTPG KPVVEPGTVL FAEVLGRTLS
Bienertia   DDFIANGKMG IVRNEDA--- --LSGQPVSG RPVVDAGTVL FAEVLGRTLS
Grape       EEFMVHGKMS IVRNDDA--- --LSRVPMQG SPVVETGTVL FAEVLGRTLS
Rice        AVASPAAGEG DRKPGERGG- ---------G RTLVPPGTVV FAEVLGRTLS
Castor      RFMVRGKIGI IKDEAAMSG- --GP---GLG RPVVETGTIL FAEVLGRTLS
Barley      EFAAEGRVGV AVGGVDLKV- --GRTGKEKC APVVGVGTVV FAEVLGRTLG
Populus     -------LLG TMLTKEVLP- ------LYDK EMEFLLCDTK KDVKEFMVKG
Lettuce     EEFLMKGKMG IVRNEEA--- --LSGGPVAG QPVVEHGTVL FAEVLGRTLS
Consistency 5343225446 6643355110 0012212225 6578438886 8888888988

            .........310.........320.........330.........340.........350
Maize       GRPLLSARRL FRRLAWHRAR QILQLNEPIE VKIYEWNTGG LLTRIE----
Sorghum     GRPLLSARRL FRRLAWHRAR QILQLDEPIQ VKIYEWNTGG LLTRIE----
Arabidopsis GRPLLSSRRY FRRIAWHRVR QIKQLNEPIE VKITEWNTGG LLTRIE----
Tomato      GRPLLSTRRL FRRIAWHRVR QIKQLNEPIE VKITEWNIGG LLTRIE----
Bienertia   GRPLLSTRRY FRRIAWHRVR QMKHLGEPIE VRITEWNTGG LLTRVE----
Grape       GRPLLSTRRF FRRIAWHRVR QIKQLNEPIE VRITEWNTGG LLTRIE----
Rice        GRPLLSARRL FRRLAWHRAR QVMQLDEPIE VKIYEWNTGG LLTRIE----
Castor      GRPLLSTRRL FRRIAWHRVR QIKELNEPIE VRITEWNTGG LLTRIE----
Barley      GRPLLSARRL FRRVAWHRVR QIKQLNVPIK VKISEWNAGG LLSRIE----
Populus     KMGIVKDEVA MSPGPPGLGK PVVETGTVLF SEVLGRTLSG RPLLSTRRLF
Lettuce     GRPLISTRRL FRRLAWHRVR QIKQLNEPIE VKITEWNTGG LLTRIE----
Consistency 8889896886 8886888869 8857867897 879588878* 8888880000

            .........360.........370.........380.........390.........400
Maize       ---------- GLRAFLPKFE LVDRISSFTD LKNKVGCSIR VCIARLDEET
Sorghum     ---------- GLRAFLPKFE LVDRISSFTD LKNKVGCSIR VCIARLDEET
Arabidopsis ---------- GLRAFIPKQE LVKKVNTFTE LKENVGRRFL VQITRLNEDK
Tomato      ---------- GLRAFLPKAE LMNRVNSYTE LKENVGRRIN VLITRINEET
Bienertia   ---------- GLRAFLPKIE LVDRVNSFTE LKDKVGHWIY VQISRINEEN
Grape       ---------- GLRAFLPKAE L--------- -LNRVGRRLY VQITRIDEAK
Rice        ---------- GLRAFLPKFE LMDRINTFTD LKNKVGCSIR VCITRLDEET
Castor      ---------- GLRAFLPKAE LMNRVKNFKE LKENVSRRIN VLITRINEDN
Barley      ---------- GLRAFLPKPQ MMTRPRNFTD LKNNVGRQMH VCITKIDERT
Populus     RRLAWQRVRQ GLRAFLPKAE LMNRVNNFKE LKENVGRQIY VLIKRINESN
Lettuce     ---------- GLRAFLPKIE LVNRVNNFTE LKENVGRRIF VQITRISEDT
Consistency 0000000000 *****9**49 9647655766 8866*85573 *4*6986*56

            .........410.........420.........430.........440.........450
Maize       NDLIISEKKA WEMTYLKEGT LLQGTVCKIF PYGARVRIAG TNRSGLLHIS
Sorghum     NDLIISEKKA WEMTYLKEGT LLQGTVRKIF PYGAQVRIAG TNRSGLLHIS
Arabidopsis NDLILSEKVA WEKLYLREGT LLEGTVVKIL PYGAQVKLGD SSRSGLLHIS
Tomato      NDLILSEKEA WQMLNLQEGT LVEGTVKRLF PFGAQIRLGE TNRSGLLHIS
Bienertia   NDLILSEREA WEKSYLREGI LLEGTVRKIF PYGAQVKIGQ TNRSGLLHVT
Grape       NDLILSEKDA WEKSHLQEGT LLEGTVKKIF PYGAQIMIGE SNRSGLLHIS
Rice        NDLIISEKKA WEMTYLKEGT LLQGIVHKIF PYGAQVRIAG TNRSGLLHIS
Castor      NELILSEREA WEMLNLREGT LLEGNVRKIF PYGAQVRIGE TNRSGLLHIS
Barley      NELIISEKEA WAMLYLKEGA LLEGTVRKLF PYGAQIRIGE TNRSGLLHVS
Populus     NELILSEREA WEMINLREGT LLEGTVKKLF PYGAQVRI-- ----GETNRS
Lettuce     NDLILSEKEA WNAKHLKEGT LLEGTVRKIF PYGAQIRIGE SNRSGLLHIS
Consistency *8**8**86* *7645*7**7 *98*7*5988 *9**997964 6788*88989

            .........460.........470.........480.........490.........500
Maize       NISRGNVLSV SDILKIDDEV KVIVIKSNVP DKIAVSIADL ESAPGLFLSD
Sorghum     NITRGNVLSV SDILKIDDEV KVIVIKSNVP DKIALSTADL ESAPGLFLSD
Arabidopsis NITRRRIGSV SDVLQVDESV KVLVVKSLFP DKISLSIADL ESEPGLFISD
Tomato      NVTQAKVTSM SNLLAVDEKV KVMVVKSMFP DKISLSIANL ESEPGLFLSD
Bienertia   KISRGKFDSV SDLLAVGEKV KVLVVRSLFP EKISLSIADL ESEPGLFISD
Grape       NITRARVTSV SDLLTVDEKV KVMVVKSMFP NKIALSIADL ESEPGLFLSN
Rice        NISRGRVLSV SDILKIDEEV KVLVVKSNVP DKIALSISDL ESTPGLFLSE
Castor      NITRSRVTAV SDLLKVDERV KVLVVKSMFP DKISLSIADL ESEPGLFVSN
Barley      KITHGQLRSV SDALRVGERV KALVIKSTTP DRIALSIRDL ESEPGLFLSN
Populus     DL-------- ---LKVDEKV KVLVAKSMFP DKISLSIADL ESEPGLFVSN
Lettuce     NITRGEFASV NDVLAIDEKV KVLVV----- ---------- ----------
Consistency 7966545377 775*59786* *98*878448 6786787678 8858888685
```

Results colour-coded for amino acid conservation

```
            .........510.........520.........530.........540.........550
Maize       REKVFSEAEE MAKRYREQLP VTPQNPILDD GLPGEKLPFD --NETK----
Sorghum     REKVFSEAEE MAKRYRE--- ------QLPVM SQNPILDDGI --PGEMLPFD
Arabidopsis REKVFTEAEE MA-----K--- ------KYREK MPLVATSPIS --DRPP----
Tomato      KERVFSEAKQ MAKKFRQ--- ------NLPTV SATKKPEPLP --TDRL----
Bienertia   RQRVFAEAEE MARKYRR--- ------KLPRI SANHKSLPPP --SNLL----
Grape       KEKVFSDAEE MAKKYRQ--- ------KLPAV TATRKLEPLP --TDAL----
Rice        KARVFSEAEE MAERYRE--- ------QLPAD SKNAKLDADL P-GGTI----
Castor      KEKVFAEAEE MAKKYRQ--- ------KLPAV LATRKSATPL --SSTL----
Barley      KEKVFVEAED MARRYRE--- ------QMPHS PRSGEADSSP DDDDAV----
Populus     KEKVFAEAEE MAKKYRQ--- ------KLPAS STNLKPEIPP S-KNAL----
Lettuce     ---------- ---------- ---------- ---------- ----------
Consistency 6668857877 8855665000 0000046533 4332433323 0033340000

            .........560.........570.........580.........
Maize       ----LYANWQ WFKFLHHS-- ---------- ---------
Sorghum     DEAKLYANWQ WFKFLHHN-- ---------- ---------
Arabidopsis ----ITSSFP QGKDEEIYAN WEWFKFESQ- ---------
Tomato      ----PFEDEE NMYANWKWFK FDRDNVNME- ---------
Bienertia   --------FE DESSLYANWK WFKFLREN-- ---------
Grape       ----PFHDEA SLYANWRWFK FERDDGPN-- ---------
Rice        ----PFDDEA TLYANWKWFK FLEDGKLG-- AVTTEHNGS
Castor      ----TFDDEA TMYANWKWFK FERD------ ---------
Barley      ----PFEDEA GSYANWKWLK FIKSERADCN PSSTEPSGL
Populus     ----SSDTEA TLYANWKWFK FEKE------ ---------
Lettuce     ---------- ---------- ---------- ---------
Consistency 0000233434 2344443324 3222000100 000000000
```
