# Supplementary material for: A novel RNA binding protein affects rbcL gene expression and is specific to bundle sheath chloroplasts in C4 plants
Source: BMC Plant Biol. 2013 Sep 22;13:138. doi: 10.1186/1471-2229-13-138 (PMC3849040; doi:10.1186/1471-2229-13-138)
Supplement: Additional file 2: Figure S2 — Comparison of maize (accession # JX650053) and Arabidopsis (accession # JX843767, translated mRNA to protein) RLSB orthologs. Length: 345 (includes plastid transit sequences), Identity: 208/345 (60.3%), Similarity: 257/345 (74.5%). Protein Alignments determined on http://www.ibi.vu.nl/programs/pralinewww/. [file 1471-2229-13-138-S2.pdf]

Additional File 2: Figure S2

Results colour-coded for amino acid conservation

The current colourscheme of the alignment is for **amino acid conservation**.

The conservation scoring is performed by PRALINE. The scoring scheme works from 0 for the least conserved alignment position, up to 10 for the most co
The colour assignments are:

Unconserved 0 1 2 3 4 5 6 7 8 9 10 **Conserved**

```
            . . . . . . . . . 10 . . . . . . . . . 20 . . . . . . . . . 30 . . . . . . . . . 40 . . . . . . . . . 50
Maize       ----------  ----------  ----------  ----------  --------MP
Arabidopsis MQTLLCQPCK  SLPILTASSS  SSLIRSSGDV  RECIDFRASE  KVSKFQFHVT
Consistency 0000000000  0000000000  0000000000  0000000000  0000000053

            . . . . . . . . . 60 . . . . . . . . . 70 . . . . . . . . . 80 . . . . . . . . . 90 . . . . . . . . . 100
Maize       TPAPALP-FV  PKPQPIL---  -----HVAKA  LVPSPR---Q  TQPQARGRG-
Arabidopsis LSPFAFRGFS  ICREFAVRGA  YGIRFCSRED  VSGVGNGGIV  AEEEIELLNK
Consistency 3330*420*2  1026036000  0000002352  6212140002  4636340240

            . . . . . . . . . 110 . . . . . . . . . 120 . . . . . . . . . 130 . . . . . . . . . 140 . . . . . . . . . 150
Maize       ---LPQPVNC  ANSKRLDDAL  SAGFVRLLNA  GQVQDADSAS  G-------TC
Arabidopsis PNPLPKSENE  ESGKADDDAI  LEPFLKFFKP  EEEGEGIESE  VSDETDRVSV
Consistency 000**532*0  354*30***7  231*664443  2622641464  1000000052

            . . . . . . . . . 160 . . . . . . . . . 170 . . . . . . . . . 180 . . . . . . . . . 190 . . . . . . . . . 200
Maize       RRYDPKPGDF  AVGVVVSGTE  ARLDVAVGAD  RLATLLSKEL  LPLD----RD
Arabidopsis EYYDPKPGDF  VVGVVVSGNE  NKLDVNIGAD  MLGTMLTKEI  LPLYDKELDY
Consistency 42********  5*******4*  26***28***  3*4*7*5**7  ***0000020

            . . . . . . . . . 210 . . . . . . . . . 220 . . . . . . . . . 230 . . . . . . . . . 240 . . . . . . . . . 250
Maize       SSDPPTRQAL  --PRLGSIGV  VAGPSVDDDR  QKRG--SRTL  VAPGTVVFAE
Arabidopsis LLCDLKYDAE  EFLVNGKMGI  VKDDDEGVEI  AEFARQGRPV  VEIGTVVFAE
Consistency 2202132 4*1  00111*45*8  *332423161  3514004*36  *31*******

            . . . . . . . . . 260 . . . . . . . . . 270 . . . . . . . . . 280 . . . . . . . . . 290 . . . . . . . . . 300
Maize       VLGRTLSGRP  LLSARRLFRR  LAWHRARQIL  QLNEPIEVKI  YEWNTGGLLT
Arabidopsis VLGRTLSGRP  LLSSRRYFRR  IAWHRVRQIK  QLNEPIEVKI  TEWNTGGLLT
Consistency **********  ***6**3***  7****5***2  **********  2*********

            . . . . . . . . . 310 . . . . . . . . . 320 . . . . . . . . . 330 . . . . . . . . . 340 . . . . . . . . . 350
Maize       RIEGLRAFLP  KFELVDRISS  FTDLKNKVGC  SIRVCIARLD  EETNDLIISE
Arabidopsis RIEGLRAFIP  KQELVKKVNT  FTELKENVGR  RFLVQITRLN  EDKNDLILSE
Consistency ********7*  *1***36855  **6**44**0  342*0*4**5  *63****7**

            . . . . . . . . . 360 . . . . . . . . . 370 . . . . . . . . . 380 . . . . . . . . . 390 . . . . . . . . . 400
Maize       KKAWEMTYLK  EGTLLQGTVC  KIFPYGARVR  IAGTNRSGLL  HISNISRGNV
Arabidopsis KVAWEKLYLR  EGTLLEGTVV  KILPYGAQVK  LGDSSRSGLL  HISNITRRRI
Consistency *2***33**6  *****6***2  **4****5*6  74355*****  *****5*248

            . . . . . . . . . 410 . . . . . . . . . 420 . . . . . . . . . 430 . . . . . . . . . 440 . . . . . . . . . 450
Maize       LSVSDILKID  DEVKVIVIKS  NVPDKIAVSI  ADLESAPGLF  LSDREKVFSE
Arabidopsis GSVSDVLQVD  ESVKVLVVKS  LFPDKISLSI  ADLESEPGLF  ISDREKVFTE
Consistency 0****8*58*  64***7*8**  13****66**  *****3****  7*******5*

            . . . . . . . . . 460 . . . . . . . . . 470 . . . . . . . . . 480 . . . . . . . . . 490 . . . . . . . . . 500
Maize       AEEMAKRYRE  QLPVTPQNPI  LDDGLPGEKL  PFDNETKLYA  NWQWFKFLHH
Arabidopsis AEEMAKKYRE  KMPLVATSPI  SDRPPITSSF  PQGKDEEIYA  NWEWFKFESQ
Consistency ******6***  57*64335**  2*21112444  *1346357**  **6****133

            .
Maize       S
Arabidopsis -
Consistency 0
```
