# Supplementary material for: A novel RNA binding protein affects rbcL gene expression and is specific to bundle sheath chloroplasts in C4 plants
Source: BMC Plant Biol. 2013 Sep 22;13:138. doi: 10.1186/1471-2229-13-138 (PMC3849040; doi:10.1186/1471-2229-13-138)
Supplement: Additional file 3: Figure S3 — Genomic sequence of maize RBCL RNA S1-BINDING DOMAIN PROTIEN (RLSB) gene (GRMZM2G087628, from Maize Genomic BAC AC211368.4), with Mu inserts. Green = Exon coding regions, Blue = introns, Black = non-coding or non-transcribed sequences, *1 = rblmsb1 insert; *2 = rblmsb2 insert. ATG and TAA are in bold and underlined. [file 1471-2229-13-138-S3.pdf]

Additional File 3: Figure S3

ATGGGGTCGTGGAAAGAGTAGCAGTGTAGCACCAGACGACCCTGCCTGCATT
CGGCCCAGCCCACTCATATCCCATTCCGACAGAAAAGAAATATCGTTTCGGA
TAAAGTAACTGCACGCCTTGGCCGGCCGGAACGCCCGCAACCGACGAA**ATG**
CCGACGCCGGCGCCGGCCCTTCCTTTCGTCCCGAAGCCGCAACCCATCCTTCA
CGTCGCTAAAGCCCTGGTCCCATCCCCTCGCCAGACGCAGCCCCAGGCCCGA
GGGCGAGGCCTACCGCAGCCGGTCAATTGTGCCAACTCCAAGCGCCTCGACG
ACGCACTCTCCGCTGGCTTCGTCCGCCTTCTCAACGCCGGCCAAGTACAGGAT
GCGGACTCTGCTAGCGGAACCTGCCGGCGCTATGACCCCAAGCCGGGTGACT
TCGCGGTCGGGGTCGTAGTTTCTGGCACGGAGGCGCGCCTCGACGTTGCGGT
CGGCGCCGACCGTCTCGCCACGCTGCTCTCCAAGGAGCTTCTCCCGCTCGACC
GCGACAGTTCTGACCCGCCGACGCGACAAGCTCTGCCTCGGCTGGGGAGCAT
CGGCGTCGTGGCGGGCCCCTCCGTGGACGACGACAGACAGAAGCGCGGGAG
CAGGACGCTGGTGGCGCCCGGGACGGTAGTATTCGCTGAGGTGCTTGGACGG
ACGCTTAGCGGGCGTCCACTACT*[2]GTCGGCACGGCGGCTCTTCCGGCGCCTC
GCATGGCAC*[1]CGCGCCAGgcaggttgagtgcatttgttctcccttcaggcatttcgttattcagacatttggtgct
gatgcagtgattcatttttgtcagACAGATTCTGCAACTCAACGAGCCAATTGAGGTAAAAA
TTTACGAGTGGAACACCGGGGGGCTACTCACAAGAATTGAGGcaaggaagagtcttct
ccctacccctttctactattgatgtatcaaatggtcaccagttatgttattttgtacttatgttcatggctaagatttaggccaacttcactt
caggGTCTGAGAGCATTTCTTCCAAAATTTGAGCTCGTGGACAGGATAAGTTCA
TTTACAGACTTGAAAAATAAAGtaaggaacttttggatgtagtactgttaatgcccatttcatcaatttgattgtc
actgcttggttatgatatttgaaacagattgcgctttctggtttttttattaaataagggactgcactatctgatatttaggttaaatttcgt
ggttatgttggctgacatcacttgttgcttcttggcatataggTCGGTTGCAGCATTCGTGTGTGCATTGC
AAGGCTCGATGAAGAAACTAACGACCTGATAATTAGTGAGAAGAAAGCATG
GGttggtgcaaatacgtctctctataagctacaattctggctttgtcaataaatttacatattacatacatgttttataaattgaattgta
ctccctctatctagaaatgataatcatatttgaccagaacagaagtcaagctttataaattttgattaacaattagttaaatatattcaag
tgtagagcataaactttaaatcaatagatttgtgtttaaagtgcctctgagattatattaatttttaataattgactacatattgtaagaga
atttaagggtcaaaatcaactctgtatgatttctccttttgtcaaatacgattatcatttctggatggacgggatgatcaagctcccaaa
atactaattgtactttactgtttaccacagtcaattatgttgtcggctatttgtttgcacagctgcacttatgtacggaatgccagcatac
ttggccaaattgtcaatgataaatcaacttcttgacattgaatttgtctgcacataactaacactgcaaaagatattgtgaatttgtaat
gctgaatgtactgatgtactgaattacttatcatcagGAAATGACTTATCTTAAAGAAGGAACCCTCC
TACAAGGGACTGTTTGCAAGATTTTTCCGTATGGTGCGCGGGTTAGGATTGCT
GGGACAAACAGAaggtgtgaaatggaacctacaaggctgcatgttgttcgtgtatttacagtcaagtgtaactgcataa
gctaaaaaaactctgtttttcttattaaaccaacaaatgcaagtgggcacccaatggagtttccataccgctttgaaatctagattagt
tcatttacatggcttttgggtcaacccactgacctagagaaatcaagactcacatccctgta1maatgcaactaatagtgtctcaac
tactaatttgcAGTGGCTTACTTCACATATCGAATATTAGTCGAGGCAATGTTTTGTC
CGTAAGTGACATCCTGAAGATAGATGATGAGGTGAAAGTTATCGTGATCAAG
TCAAATGTCCCAGACAAAATTGCAGTGAggtaaacctgacctaacactagcactggctcgattcagtt
gtgaactagctgctgtgtttgtctggatcttaccgaatactttctttgtggtaaggtctcatcaacatatatgacagagttctgaaacttt
tttttttttttgctacttgatcaacaGCATAGCAGACCTTGAGAGTGCACCTGGTTTGTTTCTGT
CAGACAGAGaggtgagacagtgagttgcactccagcctagttatatatcggaatactccacctcttgtttgatgaatcttca
tcctatgtatatatgcttagtatcacttgtcttgtactgctgcAGAAAGTGTTCTCGGAAGCAGAGGAGAT
GGCAAAGAGATACAGGGAGCAGCTTCCAGTTACCCCTCAGAACCCTATATTA
GATGATGGCCTTCCAGGAGAAAAGCTACCATTTGATAACGAAACTAAACTGT

ATGCAAACTGGCAATGGTTCAAATTTCTGCATCATAGC**TAA**GCTGCTGACAA
CAGCGACAGAGACATATCTGGTACTTGTTGACCATGCAAAGGTGAACTGTCG
CTAGAGAACCTGCCAACCTGTTGAAGTCCAATACTGACCAGTGGTGGGTAGT
TCTCCACTTCCATAACCCAGCATCAGTCATGGAGAAGTATGTTGGACAGGAA
CAGGCAACTTCATCCCGCAGACAAGATAGGGTACAAGGTTTCCAGGCTGCAG
AACATTGGTGTTTCGAATTGCCTCATTCCTAGTGCCCCTGGAGTGAAATGTTG
TGATATCATTTTATCTGTCCGGAGAATGTAGCTTCATTGCATTCATGTTATGTC
AAAACTAGTCGGGCTGCCCAAGCACTCCGTGCCAACAACTTGCATCTGCCCA
TGAAATAGATTCTGAAATTTTAGCACCGTGTCCCTGGATTGCTGAAGTATCGT
GATGGGCGCAAGGGAGTACCTGAATTATTATGTACACTCTACCTGGCTAGTC
ACAACTGGCAGTCCTGTTAGACAAAATTGGAGTCCAGGAAAAGAGGAGGAA
GATTATGGGCTTGGCCCCTGCCCAGCCGGTTTGCCAAAAGCCAGTTGCCCTCT
AAGTATAGATGTCCAAATGGGTAGACCGTGCCG
