# Supplementary figures and images for: A novel RNA binding protein affects rbcL gene expression and is specific to bundle sheath chloroplasts in C4 plants
Source: BMC Plant Biol. 2013 Sep 22;13:138. doi: 10.1186/1471-2229-13-138 (PMC3849040; doi:10.1186/1471-2229-13-138)

## Additional File 4: Figure S4

**Top panels:**

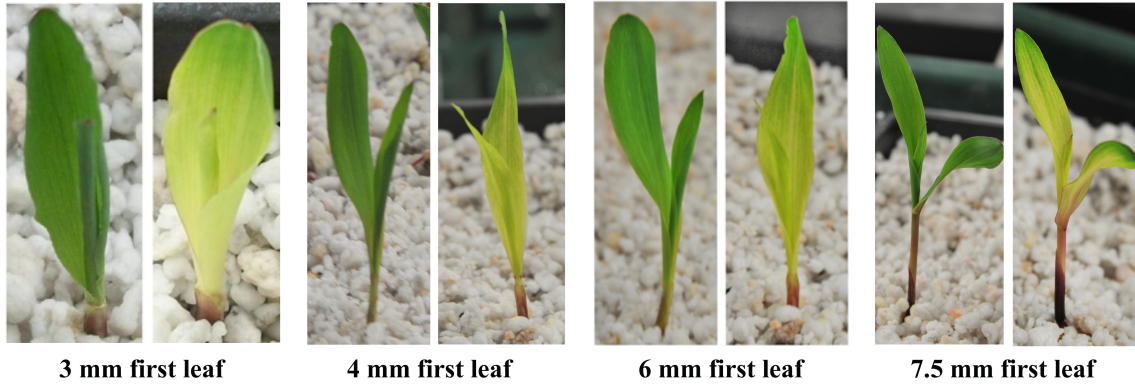

**Bottom panels:**

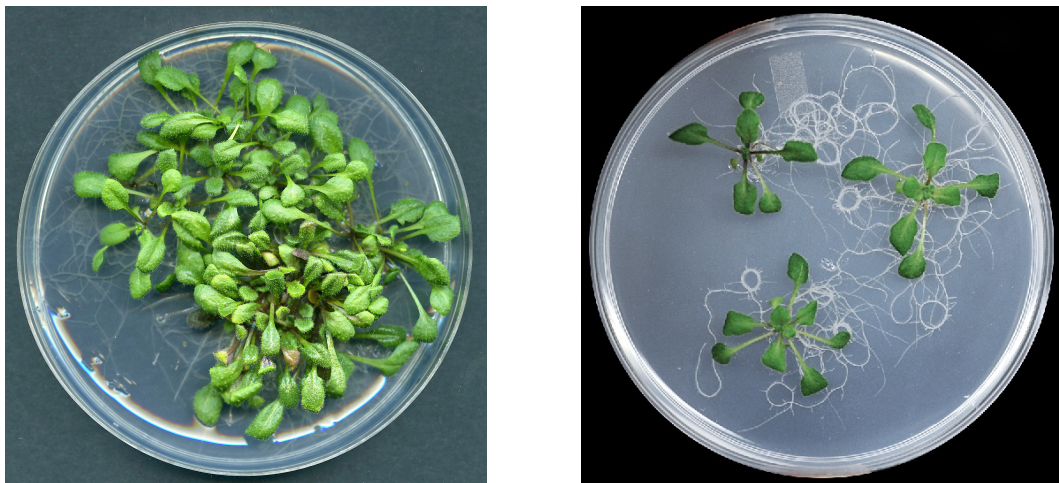

Supplement: Additional file 4: Figure S4 — Top panels: Mu-insertion mutants within the RLSB gene of maize cause a virescent (pale) yellow phenotype in maize seedlings. (Left top panels) Non-mutant RLSB/RLSB seedlings. (Right top panels) Double insertion-mutant rlsb-1/rlsb-2 seedlings. Sizes in mm indicate the length of the fist leaf on a mutant plant. Note that each image pair is shown at a different scale. Bottom panels: Wild type Col0 Arabidopsis plants growing on MS media supplemented with increased sucrose. A stepwise increases of 3% (left) to 8% (right) sucrose was necessary to initiate and support the growth of the low Rubisco rlsb-silenced plants, but had no visible effects on the non-transformed Col0. Note that the plants shown in this figure are six weeks old, slightly younger than the two rlsb-silenced plants shown in Figure 8. [file 1471-2229-13-138-S4.pdf]

Additional File 5: Figure S5

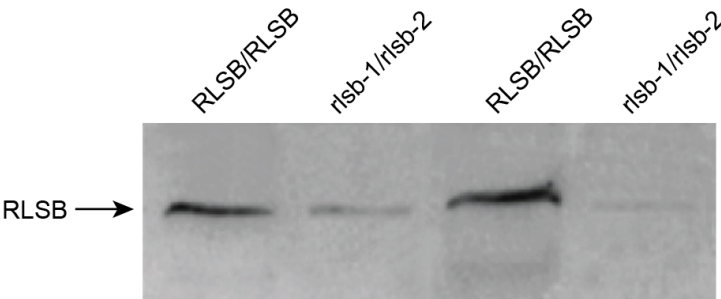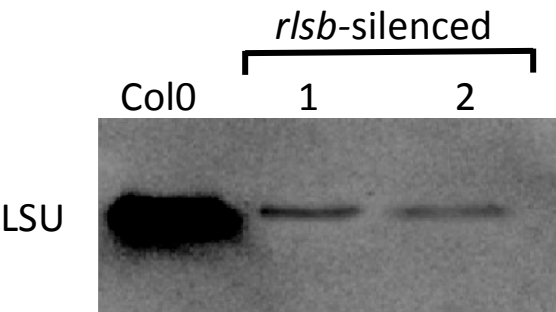

Supplement: Additional file 5: Figure S5 — Long digital exposures (using ImageQuant Software) of selected western blots. Top: RLSB immunoblot blot of Figure 5A, middle panel. This enhanced, longer exposure image shows very low levels of RLSB protein accumulating in two of the rlsb-1/rlsb-2 insertion mutants. Note that the lower level of RLSB in the second double mutant (lane 4), relative to the first double mutant (lane 2) corresponds to a lower level of Rubisco LSU in the same mutant, shown in the corresponding LSU lane of Figure 5, panel A. Bottom: Immunoblot of LSU Figure 8, panel C, LSU. This digitally enhanced exposure image shows that very low levels of LSU protein accumulation in two silenced Arabidopsis plants (S1 silenced). This digitally enhanced exposure image shows that very low levels of LSU protein accumulation in two silenced Arabidopsis plants (S1 silenced). [file 1471-2229-13-138-S5.pdf]

Additional File 6: Figure S6

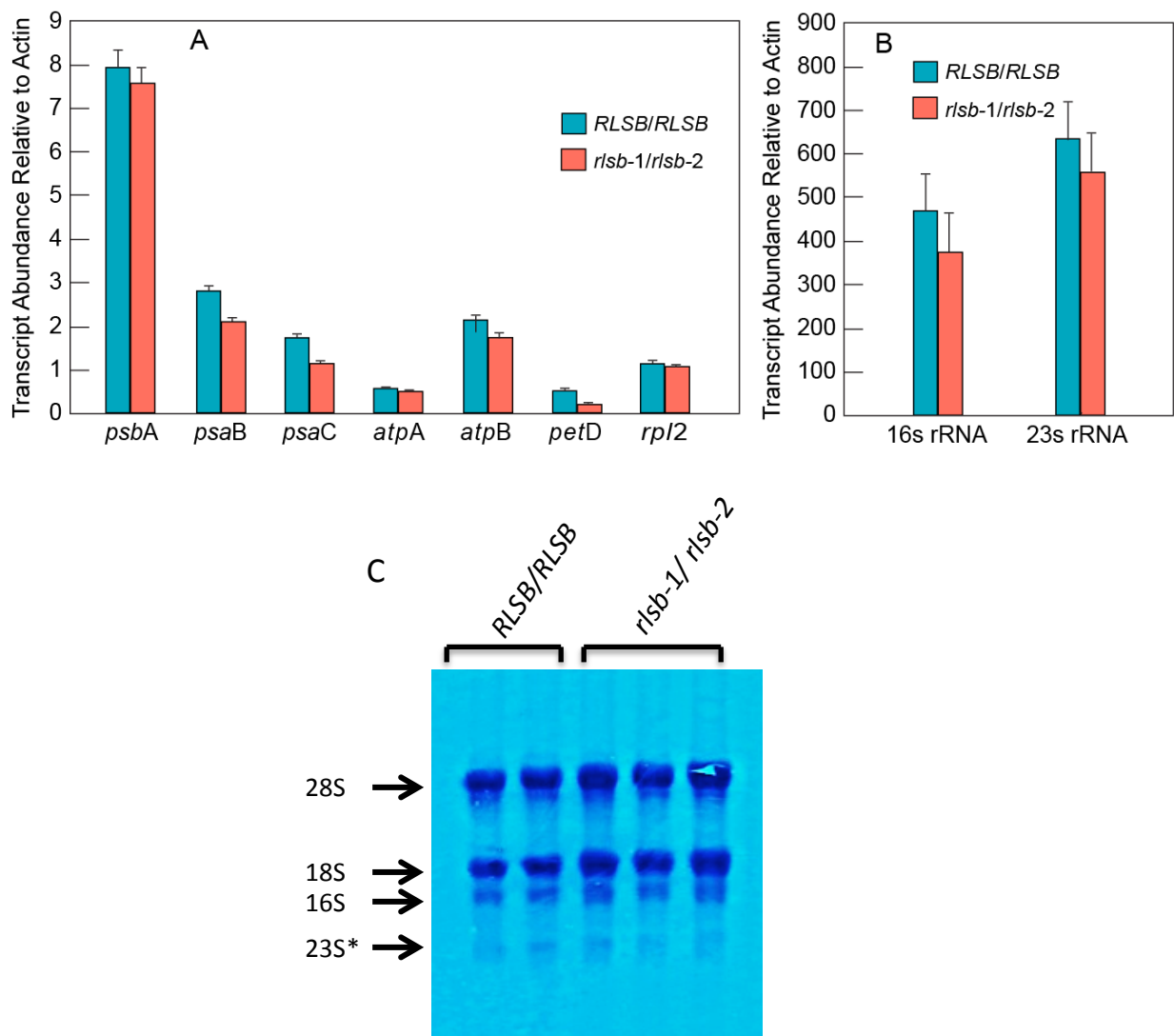

Supplement: Additional file 6: Figure S6 — Accumulation of plastid-encoded mRNA and rRNA in lower leaf regions from RLSB/RLSB and rlsb-1/rlsb-2 maize seedlings. A. Accumulation of several plastid-encoded mRNAs. B. Accumulation of plastid-encoded rRNAs. C. Formaldehyde-agarose gel, transferred to nitrocellulose and stained with methylene blue, showing cytoplasmic and chloroplast rRNAs in two RLSB/RLSB plants and three rlsb-1/rlsb-2 mutant plants. 28S and 18S are cytoplasmic rRNAs. 16S rRNA and the 23S* cleavage product of 23S rRNA are chloroplastic. For qRT-PCR shown in A and B, quantification of transcript levels was standardized to actin mRNA. Data is averaged for two RLSB/RLSB plants and four rlsb-1/rlsb-2 siblings, with three repeats run for each of the plant samples. Note differences in scale for panels A and B, due to the much greater abundance of rRNA relative to the mRNAs. Statistical significance was calculated using Student’s t-test. For each bar, P values were less than 0.05. [file 1471-2229-13-138-S6.pdf]

Additional File 7: Figure S7

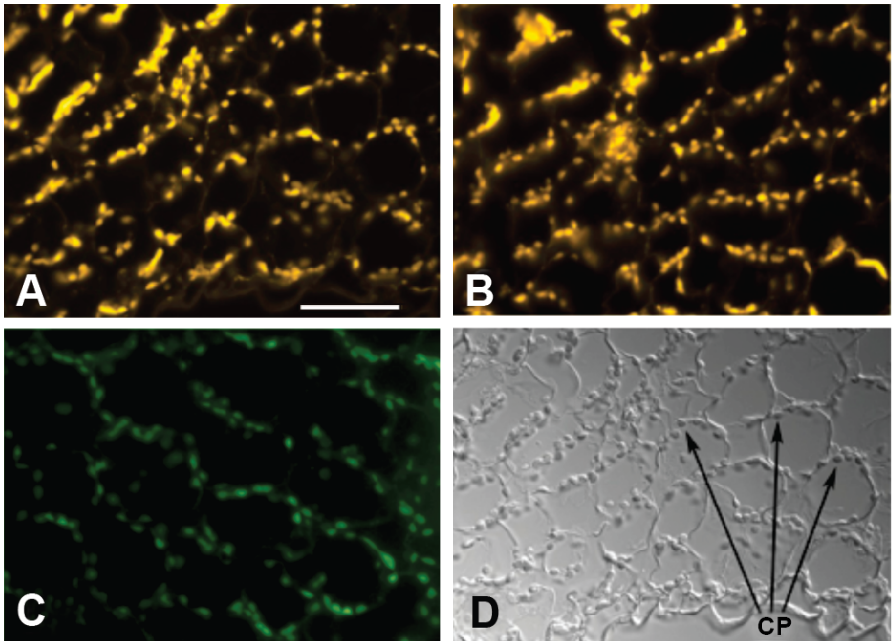

Supplement: Additional file 7: Figure S7 — Low magnification immunolocalization image of RLSB and Rubisco LSU proteins in leaf sections of the C3 plant Arabidopsis. A. Arabidopsis leaf section reacted with RLSB primary antiserum. B. Arabidopsis leaf sections reacted with LSU primary antiserum. C. Arabidopsis leaf section showing autofluorescence of plastids (imaged enhanced) from a section reacted with secondary antibody alone. D. DIC image of the images shown in A and C, with chloroplasts indicated. cp, chloroplasts. Arabidopsis leaf sections were incubated with the indicated primary antiserum, and then with R-phycoerythrin (A, B) conjugated secondary antibody. Images were captured using a 20X objective of a Leica DMIRE2 inverted fluorescent microscope, bar = 100 μM. [file 1471-2229-13-138-S7.pdf]

**Additional File 8: Figure S8**

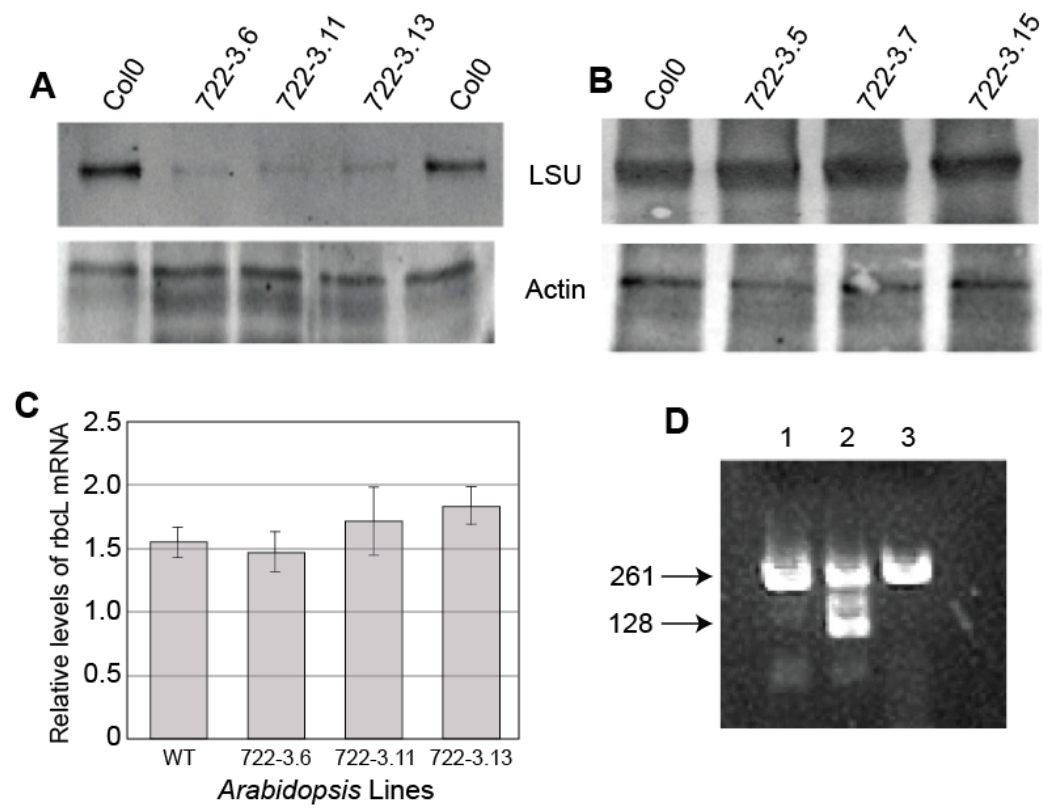

Supplement: Additional file 8: Figure S8 — Rubisco protein and mRNA accumulation in rlsb T-DNA insertion heterozygotes, non-mutant siblings, and wild type Col0 Arabidopsis. A. Western analysis. Total protein from heterozygous SALK_015722 containing a T-DNA insert in one copy of the RLSB locus, and wild type non-insert containing plants reacted with LSU antisera (top panels). Equal amounts of protein were loaded in each lane. As a control, the blot was re-probed with actin antisera (bottom panels). B. Segregating wild type siblings lacking T-DNA inserts showed no LSU reduction. C. qRT-PCR analysis of rbcL mRNA in wild type and SALK_015722 heterozygotes. For each plant, random primers produced cDNA from mRNA; these were then incubated with primers for Arabidopsis rbcL mRNAs, or for plastid-encoded rps3 and rpl20 (standardization controls). For each bar, P values were less than 0.05. D. Genomic PCR analysis of T-DNA insert in At1g71720 locus. Ethidium-bromide stained agarose gel showing representative PCR amplifications using total DNA isolated from the indicated plants. The three primers added to each PCR reaction were: LP (At1g71720 sequence upstream of insert site) = TCGATTGCTGATTTTGATTCC; RP (At1g71720 sequence downstream of insert site) = TTCCTTCCCCTTTTTCATGTC; LBB1 (left border of T-DNA) = GCGTGGACCGCTTGCTGCAACT (Reverse AGTTGCAGCAAGCGGTCCACGC). Sequencing confirmed the identity of the amplified fragments. A 261 nucleotide band corresponding to wild type At1g71720 was amplified from LP and RP primers if there was no insert; A 128 nt band was amplified from LP and LBB1 if the locus contained an insert. Lane 1 = wt Col0 (normal LSU levels), showing a band of 261 nt. Lane 2 = heterozygote AT SALK 017226 (reduced LSU), showing two amplified bands of 261 and 128 nt. Lane 3 = AT SALK 017226 segregate (sibling plant with normal LSU levels and no T-DNA insert), showing a single amplified band at 261 nt. [file 1471-2229-13-138-S8.pdf]
