# Supplementary material for: A novel RNA binding protein affects rbcL gene expression and is specific to bundle sheath chloroplasts in C4 plants
Source: BMC Plant Biol. 2013 Sep 22;13:138. doi: 10.1186/1471-2229-13-138 (PMC3849040; doi:10.1186/1471-2229-13-138)
Supplement: Additional file 9 — List of primer sequences used for this study. [file 1471-2229-13-138-S9.pdf]

Additional File 9: List of Primer sequences used for this study.

*__Primers for amplification of full-length *RBCL RNA S1-BINDING DOMAIN PROTIEN (RLSB) cDNA*:__

Arabidopsis RLSB forward: ATGCAAACGCTTCTCTGTC
Arabidopsis RLSB reverse: TGTCACTGACTCTCGAAC
Maize *RLSB* forward: ATGCCGACGCCGGCGCCGGCCCTTC
Maize *RLSB* reverse: GCTAAAATTTCAGAATCTATTTCATGG

*__Primers for amplification of 447 nt fragment of *RLSB* for pEt17b and pHannibal__

For pEt:

At249NdeForward: GTATACATATGGTTGGAAATGGAGGAATCGTGG underline = Nde1 site
At696NheReverse: ATTATGCTAGCCTCCGCAATCTCAACACC underline = Nhe1 site

For pHannibal:

At249RiXbaForward: GTATATCTAGAGTTGGAAATGGAGGAATCGTGG underline = Xba1 site
At249RiXhoFoward: GTATACTCGAGGTTGGAAATGGAGGAATCGTGG underline = Xho1 site
At696RiClaReverse: ATTATATCGATCTCCGCAATCTCAACACC underline = Cla1 site
At696RiKpnReverse: ATTATGGTACCCTCCGCAATCTCAACACC underline = Kpn1 site

*__Primer Sequences for genomic screening of Mu insertion library:__

tk1314S13': CGAGCCTTGCAATGCACACA (maize genomic)
tk279S15': CCGGTCAATTGTGCCAACTC (maize genomic)
eomu1: GCCTCCATTTCGTCGAATCCC (Mu border)
eoMu2: GCCTCTATTTCGTCGAATCCC (Mu border)

*__Primer sequences for quantative real time PCR (qRT-PCR):__

Maize *rbc*L forward: AGTGTCTACGCGGTGGACTTGATT
Maize *rbc*L reverse: TGCAGTCGCATTCAAGTAATGCCC
Maize *RLSB* forward: ATTGCTGGGACAAACAGAAGTGGC
Maize *RLSB* reverse: AAACCAGGTGCACTCTCAAGGTCT
Maize *actin* forward: TGTGTTGGACTCTGGTGATGGTGT
Maize *actin* reverse: TGATGTCCCGTACGATTTCCCGTT
*Arabidopsis rbc*L forward: AGTGTCTACGCGGTGGACTTGATT
*Arabidopsis rbc*L reverse: TGCAGTCGCATTCAAGTAATGCCC
Arabidopsis RLSB forward: TGGTGGAGATTGGGACGGTTGTTT
Arabidopsis RLSB reverse: TCAATTCTGGTGAGAAGCCCTCCA
Arabidopsis actin forward: ACACTGTGCCAATCTACGAGGGTT
Arabidopsis actin reverse: ATTTCACGCTCTGCTGTTGTGGTG
Arabidopsis rps3 forward: AGGAACTCTGCCTTCTCTGATCCA
Arabidopsis rps3 reverse: TCTTGCAGAATTTATCGCTGGAC
Arabidopsis rpl20 forward: CCCATTTCGTGAATTACGGCATTT
Arabidopsis rpl20 reverse: GCTCATTCACGGCTTACACGAACT

*__Primer sequences for RNA immunopurification with quantitative real-time PCR (RIP/qRT-PCR)__

*rbc*L forward: GCTGCGGAATCTTCTACTGG
*rbc*L reverse: GTAGAGCGCGTAAGGCTTTG
*psa*B forward: GTCCTGTCCAAGCCAAAGAA

*psa*B reverse: TGGGGTTTATCAGTGGTGGT
*psb*A forward: TCGCTGCTCCTCCAGTAGAT
*psb*A reverse: CTCATAAGGACCGCCATTGT
*pet*D forward: CCAACGGGCTTATTGACAGT
*pet*D reverse: GCGGTGGAATGAATCAACTC
*psa*C forward TCAATAAGATAGAGCCATGCTG
*psa*C reverse AGGATGCACTCATTGTGTACGA
*atp*A forward GACAGACTGGCAAAACAGCA
*atp*A reverse CCGTTCGCGGTACATAAAAT
*atp*B forward CACCGACTGGAACACTGAGA
*atp*B reverse GACCCGTGCTGGATATCACT
*rpl*2 forward: GCCGTATGCTTTGGAAGAAG
*rpl*2 reverse: GGACCTCCCCAGATGGTAAT
